# Supplementary material for: A systematic review of self-report measures used in epidemiological studies to assess alcohol consumption among older adults
Source: PLoS One. 2021 Dec 16;16(12):e0261292. doi: 10.1371/journal.pone.0261292 (PMC8675766; doi:10.1371/journal.pone.0261292)
Supplement: S1 Table — (DOCX) [file pone.0261292.s002.docx]

**S2 Table: Self-report measures used in epidemiological studies to assess alcohol consumption among older adults**

| **Author**  **Year**  **Country** | **Aim of the study** | **Participants: numbers, gender, age** | **Longitudinal studies**  **Data**  **Sample** | **Method** | **Definition and measure** |
| --- | --- | --- | --- | --- | --- |
| Agahi et al.  2016 [1]  Sweden | Association between alcohol consumption and survival | N = 863  Included both women and men  ≥ 76 years  Age range 76-101 | Longitudinal, prospective study.  Data from the fourth wave of the Swedish Panel Study of Living Conditions of the Oldest Old (2010/2011).  Nationally representative sample. Institutionalized and non-institutionalized individuals. Face-to-face interview, proxy inter-views or questionnaire sent by mail. | Self-reporting.  Assessed drinking frequency last 12 months.  Assessed quantity: number of drinks consumed on a typical drinking day.  Calculated the number of drinks consumed per month.  Did not define alcohol content (g) in one drink. | Abstainer: Did not drink alcohol  Light-to-moderate drinkers: 0.5-30 drinks/month  Heavy drinkers: > 30 drinks/month |
| Agahi et al.  2019 [2]  Sweden | Association between social integration and patterns of drinking frequency in older adults | N = 1,043  ≥ 65 years  Mean age women:  75.7 (± 7.2)  Mean age men:  74.0 (± 6.4) | Longitudinal, prospective study.  Data from The Swedish Level of Living Survey (2010) and Swedish Panel Study of Living Conditions of the Oldest Old (2011 and 2014). Study setting and participants not clearly described. Face to face interview. | Self-reporting.  Assessed drinking frequency last 12 months. | Monthly or less  Every week:  1-4 times/week  Daily or almost daily:  5-7 times/week |
| Aguila et al.  2016 [3]  Mexico | The relationship between sociodemographic characteristics and alcohol use among low-income older adults | N = 2,351  52-53% women  ≥ 70 years  Mean age 78-79 (± 6) | Longitudinal, prospective study.  Data from three waves of regional studies in Mexico (2008-10). Randomly selected sample. Community dwelling older adults. In-person interview. | Self-reporting.  Assessed lifetime use of alcohol, current use of alcohol, weekly drinking frequency last month, and quantity: number of drinks consumed on drinking days last month.  Did not define alcohol content (g) in one drink. | Weekly drinking frequency:  ≥ 2 days/week  Drinks per day  ≥ 3 drinks/day  Problematic alcohol use:  > 1 standard drink/day or  > 7 standard drinks/week and  > 3 drinks on one occasion. |
| AlGhatrif et al.  2013 [4]  USA | Association between alcohol consumption and mortality among older Mexican American men | N = 908  100% men  ≥ 65 years  Mean age: 71.1 (± 4.1)  Age range: 65-80 | Longitudinal, prospective study.  Data from five waves of the Hispanic Established Population for the Epidemiologic Study of the Elderly (1993-94, 1995-96, 1998-99, 2000-01, 2004-05). Community-dwelling men. In home interviews. | Self-reporting.  Assessed ever use of alcohol and any alcohol use during the past year and the past month. Assessed drinking frequency past month and how many drinks on average consumed per occasion. Calculated the number of drinks consumed last month.  Did not define alcohol content (g) in one drink. | Lifetime abstainers: Not drinking alcohol in their entire life.  Former drinkers: Drunk alcohol during their entire life, but not the past month.  Low risk drinkers: ≤ 30 drinks during the month prior to the interview and ≤ 3 drinks/occasion.  At-risk drinkers: > 30 drinks during the month prior to the interview or > 3 drinks/occasion. |
| Almeida et al.  2014 [5]  Australia | Association between alcohol consumption and cognitive impairment in older men | N = 3,542  100% men  ≥ 65 years  Mean age: 71.3 (± 4.1)  Age range: 65-83 | Longitudinal, prospective study.  Collected during 2001 to 2004.  Community-dwelling men. | Self-reporting.  Assessed alcohol consumption during the past year.  Calculated the total number of drinks consumed during a usual week.  1 standard drink = 10 g of alcohol. | Abstainers  Occasional drinkers  Regular drinkers: < 15 drinks/week  Moderate drinkers: 15-27 drinks/week  Heavy drinkers: 28-34 drinks/week  Abuse: ≥ 35 drinks/week |
| Almeida et al.  2017 [6]  Australia | Association between excessive alcohol consumption and mortality in older men | N = 3,496  100% men  ≥ 70 years  Mean age: 77.1 (± 3.6)  Age range: 70-89 | Longitudinal, retrospective study.  Data from the Health of Men Study (1996-98, 2001-04). Community-representative cohort. | Self-reporting.  Assessed ever use of alcohol, and any alcohol use during the preceding year. Assessed number of drinks consumed each day of a usual week.  Calculated the average number of drinks consumed per day. 1 standard drink = 10 g of alcohol. | Never drinkers  Past drinkers  ≤ 2 drinks/day  2-4 drinks/day  4-6 drinks/day  > 6 drinks/day: regular excessive alcohol consumption |
| Bazal et al.  2019 [7]  Spain | Association between alcohol consumption and atrial fibrillation | N = 6,527  Included both women and men.  ≥ 55 years  Mean age: 65-69  Age range: 55-80 | Longitudinal study.  Data from the PREDIMED study (2003). Participants from 11 recruiting centers. Study setting, and participants not clearly described.  Mean follow-up 4.4 years. | Self-reporting.  Used a validated 137-item food-frequency questionnaire to measure alcohol consumption at baseline and every year.  Assessed the frequency of the consumption of different alcoholic beverages (50 cc for liquor or spirits, 100 cc for wine and 330 cc for beer).  Daily alcohol consumption was estimated by adding all different beverages.  Recall period not reported.  1 unit = 10 g alcohol | Frequency of consumption of different alcoholic beverages:  Never  1-3 monthly  Three categories for weekly consumption: 1; 2-4; 5-6  Four categories for daily consumption: 1; 2-3; 4-6; > 6  Non-drinkers  Mediterranean alcohol drinking pattern: 10-30 g/day in men and 5-15 g/day in women, preferably red wine consumption with low spirits consumption.  Low-moderate drinking: < 30 g/day in men and < 15 g/day in women.  Heavy drinking: ≥ 30 g/day in men and ≥ 15 g/day in women.  Classification according to units and grams of alcohol:  Abstainers  Low-moderate drinkers: ≤ 70 g/week in women (> 0  and ≤ 7 units/week) and ≤ 140 g/week in men (> 0  and ≤ 14 units/week).  Heavy drinkers: > 70 g/  week (>7 units/week) in women and > 140 g/week (> 14 units/week) in men. |
| Bell et al.  2015 [8]  England | Reliability of alcohol consumption in older adults based on the AUDIT-C | N = 5,980  28% women  Mean age: 69.7 (± 5.8)  Age range 59-82 | Longitudinal, retrospective study.  Data from the Whitehall II prospective cohort study (1985-2013). Community dwelling adults. | Self-reporting.  Assessed drinking frequency in the past 12 months. Assessed alcohol consumption by the tool AUDIT-C (3 questions: drinking frequency, quantity, binge drinking).  Did not define alcohol content (g) in one drink. | Drinking frequency  never  special occasions  once a month or less  2-3 times per week  4+ times per week  Number of drinks per drinking day  1-2 drinks  3-4 drinks  5-6 drinks  7-9 drinks  10+ drinks  Hazardous drinking:  Men: AUDIT-C score ≥ 4  Women: AUDIT-C score ≥ 3 |
| Britton et al.  2020 [9]  United Kingdom | Association between alcohol consumption and sleep disorders  among older adults | N = 6,117  29% women  Mean age women: 69.6  Mean age men: 69.4 | Longitudinal study.  The Whitehall II study.  Phases 1, 3, 5, 7, 9, 11 (1985-2013). Followed for 30 years.  Self-completed questionnaire. Community dwelling adults (civil servants). | Self-reporting.  Assessed number of drinks consumed last 7 days. Drinks converted into UK units of alcohol. 1 UK unit = 8 g alcohol. Converted units (spirits, wine, beer) were summed to define the total number of UK units consumed.  Assessed a retrospective alcohol life-course grid with the use of AUDIT-C: drinking frequency, number of drinks on a typical  drinking day, and frequency of consuming ≥ 6 drinks in a single occasion. | Non-drinker: did not drink alcohol last year.  Former drinkers: previously reported consumption but none in the most recent phase.  Moderate: within UK guidelines: 1–14 (8–112 g) units/week.  Heavy: above UK guidelines: ≥ 15 units/week.  Hazardous drinking: AUDIT-C score ≥ 5  Alcohol volume per week:  Non-drinker  None in the past week  1-14 units  14-21 units  > 21 units  Typologies of alcohol consumption over measurement periods:  1) Stable none  2) Stable moderate  3) Stable heavy  4) Unstable moderate (at least half of the phases were moderate)  5) Unstable heavy  6) Former drinker  Hazardous drinking: AUDIT-C score ≥ 5 on ≥ 3 data collection phases. |
| Buja et al.  2011 [10]  Italy | Association between alcohol consumption and renal impairment in older adults | N = 3,404  47.6% women  ≥ 65 years  Age range 65-84 | Longitudinal, retrospective study.  Data from the Italian Longitudinal Study on Aging (1992-1995).  Representative sample. Study setting, and participants not clearly described. Face-to-face interview. | Self-reporting.  Assessed abstainers, former, and current drinkers.  Assessed the frequency of drinking wine, beer, or spirits.  Assessed number of intake of glasses of wine or beer per day, and number of shots per day, month, or year. Standard measure or a glass of wine and beer was 125 ml. Standard measure of a shot: 40 ml. The quantities of beverages were converted into ml/day, and total consumption (g/day) was calculated by multiplying the beverages consumed by their alcohol content. | Abstainers: No current or past alcohol consumption.  Former drinkers: Consumed alcohol in the past, but no longer at the time of the interview.  Current drinkers: Consumed alcohol at the time of the interview.  Current consumption: The amount reported at the time of the interview.  Volume consumed in men:  ≤ 12, 13-24, 25-47, ≥ 48 g/day  Volume consumed in women:  ≤ 12, 13-24, > 24 g/day |
| Buja et al.  2010 [11]  Italy | Association between alcohol consumption and metabolic syndrome in older adults | N = 3,627  47.7% women  ≥ 65 years  Age range 65-84 | Longitudinal, prospective study.  Data from the Italian Longitudinal Study on Aging (1992-1996). Multicenter study on a population-based sample. Study setting, and participants not clearly described. Face-to-face interview. | Self-reporting.  Assessed number of glasses per day of wine or beer and glasses per week of spirits. One glass assumed to be 125 ml. The quantities of beverages were converted into ml/day, and total consumption (g/day) was calculated by multiplying the beverages consumed by their alcohol content.  Recall period last week. | Abstainers  Volume consumed in men:  ≤ 12, 13-24, 25-47, ≥ 48 g/day  Volume consumed in women:  ≤ 12, 13-24, > 24 g/day |
| Chavez et al.  2016 [12]  USA | Association between unhealthy alcohol use and rehospitalization in older adults | N = 579,330  0.3-1.9% women  Veteran Affairs patients ≥ 65 years  Mean age 77 (± 7.4) | Longitudinal, retrospective study.  Included Veteran Affairs patients if they were hospitalized in 2009-2011 and had at least one AUDIT-C score in their medical record in the year before hospitalization.  Community-dwelling older adults. Face-to-face interview. | Self-reporting.  Assessed risky alcohol use with the screening tool AUDIT-C (3 questions).  Score 0-12 points. Recall period 12 months. | Four AUDIT-C categories:  Non-drinking: Score 0  Low-risk drinking:  Men: AUDIT-C score 1-3  Women: AUDIT-C score 1-2  Moderate-risk drinking:  Men: AUDIT-C score 4-7  Women: AUDIT-C score 3-7  High-risk drinking:  Men/women: AUDIT-C score 8-12 |
| Cohen-Mansfield et al.  2012 [13]  Israel | Trends in health behaviors in older adults | N = 1,621  Cohort 1: 44.9% women  Cohort 2: 42% women  ≥ 75 years  Age range 75-94 | Longitudinal, prospective study.  Cohort 1: Cross-sectional and Longitudinal Aging Study (1989-92).  Cohort 2: The Israeli Multidisciplinary Aging Study (2000-02).  Representative samples. Community dwelling older adults.  In-home interview. | Self-reporting.  Assessed type of alcoholic beverage during the previous month. Assessed the frequency of drinking and the number of drinks during each drinking episode.  Did not define alcohol content (g) in one glass of different beverages. | Number of glasses of wine per day:  0, 1, > 1  Number of glasses of beer per day:  0, 1, > 1  Number of glasses of alcoholic beverages other than wine or beer:  0, 1, > 1 |
| Dhana et al.  2020 [14]  USA | Association between healthy lifestyle and the risk of Alzheimer  Dementia | N = 2,765  62.4-75.2% women  Mean age: 73.2-81.1 (± 5.8-7.2) | Longitudinal study.  Data from the Chicago Health and Aging Project (follow-up 5.8 years) and the Rush Memory and Aging Project (follow-up 6.0 years).  Community dwelling older adults. | Self-reporting.  Used the food frequency questionnaire to assess average frequency of intake of wine and other alcoholic beverages.  Did not define alcohol content (g) in one drink.  Recall period not reported. | Low risk (light to moderate alcohol consumption):  Women: ≥ 1 - < 15 g/day (up to 1 drink a day)  Men: ≥ 1- < 30 g/day (up to 2 drinks a day)  High risk:  Women: ≥ 15 g/day  Men: ≥ 30 g/day |
| Gargiulo et al.  2013 [15]  Italy | Association between alcohol consumption and mortality in older adults with chronic heart failure | N = 1,187  56.5% women  ≥ 65 years  Mean age 74.4 (±6.4) | Longitudinal, prospective study.  Data from the “Osservatorio Geratrico Campania study” (1992-2003). Random sample.  Community dwelling older adults with and without chronic heart failure.  In-home interview. | Self-reporting.  Assessed alcohol intake in ml per day. Included participants who drank wine. Asked if they drank ¼ liter, ½ liter, 1 liter, or more than 1 liter. | Non-drinkers  Stratified drinkers into:  ≤ 250 ml/day  > 250 - ≤ 500 ml/day  > 500 - ≤ 1000 ml/day  > 1000 ml  Moderate drinkers:  ≤ 250 ml/day |
| Goulden  2016 [16]  USA | Association between alcohol consumption and mortality among middle-aged and older adults | N = 24,029  58% women  ≥ 50 years  Mean age 65.9 (± 10.8) | Longitudinal, prospective study.  Data from the Health and Retirement study (1995-2012). Nationally representative sample. Study setting, and participants not clearly described. Telephone or face-to-face interview. | Self-reporting.  Assessed average weekly alcohol consumption for the last 3 months. Assessed number of drinks per week.  Did not define alcohol content (g) in one drink. | Non-drinkers: Being abstinent from alcohol each time over a period of 4 years.  Occasional drinkers:  Drinking on at least once over a period of 4 years, but less than once a week.  Regular drinkers: Dinking at least 1 drink/week on at least 1 occasion (over a period of 4 years).  Drinks/week in regular drinkers:  < 7, 7 to < 14, 14 to < 21, ≥ 21 |
| Halme et al.  2010 [17]  Finland | Alcohol consumption and mortality in older adults | N = 1,569  65.3% women  ≥ 65 years  Mean age men:  72.7 (± 6.02).  Mean age women:  74.7 (± 6.7) | Longitudinal, prospective study.  Data from the Health 2000 Study (2000-2001).  Population-based survey. Community-dwelling older adults. In-home interview. | Self-reporting.  Assessed alcohol consumption by beverage-specific quantity and frequency questions over a 12-month period.  The beverage-specific amounts were converted to Finnish standard drinks by multiplying the weekly quantities and weekly frequencies.  1 standard drink = 12 g of alcohol. | Abstainer: Not drinking at all during the last year.  Moderate drinking in two categories.  Moderate drinking: < 1 drink/week  Moderate drinking: 1-7 drinks/week  Heavy drinking in two categories.  Heavy drinking: 8-14 drinks/week  Heavy drinking: ≥ 15 drinks/week |
| Hassing  2018 [18]  Sweden | Association between alcohol consumption and cognitive function in older adults | N = 486  64% women  ≥ 80 years  Mean age 83 (± 2.6) | Longitudinal, prospective study.  Data from the Swedish Twin Registry (1967) and the OCTO-Twin study (1991-93). Community dwelling adults. | Self-reporting.  Assessed ever use of alcohol, types of beverages consumed, drinking frequency and how much they drank on a typical occasion. Estimated units/week.  1 unit = 12 g of alcohol.  Recall period not reported. | No alcohol intake: 0 units/week  Alcohol intake: mean units/week  Occasional: < 1 drink/week  Low: 4 drinks/week  Moderate: 8 drinks/week |
| Heffernan et al.  2016 [19]  Australia | Association between alcohol consumption and dementia in older adults | N = 821  55.3% women  Mean age 78.6 (±4.8)  Age range 70-90 | Longitudinal, prospective study.  Data from the Sydney Memory and Ageing Study (year not reported). Non-demented community dwelling older adults.  Face-to-face interview. | Self-reporting.  Assessed alcohol consumption last 12 months, frequency of alcohol consumption and the number of drinks per drinking session. Estimated the number of drinks consumed per day.  In Australia 1 standard drink = 10 g alcohol  In USA 1 standard drink =  14 g of alcohol. | Abstainers:  No alcohol in past 12 months  **Using Australian guidelines**:  Low risk:  Men: ≤ 4 drinks/day  Women: ≤ 2 drinks/day  Risky  Men: > 4 drinks/day  Women: > 2 drinks/day  **Using US guidelines**  Low risk:  Men: ≤ 4 drinks/day and ≤ 14 drinks/week  Women: ≤ 3 drinks/day and ≤ 7 drinks/week  Increased risk  Men: > 4 drinks/day or > 14 drinks/week  Women: > 3 drinks/day or > 7 drinks/week  Highest risk.  Men: both > 4 drinks/day and > 14 drinks/week  Women: both > 3 drinks/day and > 7 drinks/week |
| Hoang et al.  2014 [20]  USA | Association between alcohol consumption and cognitive impairment in older women | N = 1,309  100% women  ≥ 65 years  Mean age 68.3 (±2.8)  Age range 65-81 | Longitudinal, prospective study.  Data from the Women Cognitive Impairment Study of Exceptional Aging (1986-88).  Community dwelling women. | Self-reporting.  Assessed frequency and amount (number of drinks on each occasion) of alcohol consumed in the past 30 days. Average number of drinks per week was calculated. One drink was defined as one 12 oz beer, one 5 oz glass of wine, or one drink with 1-1.5 oz of liquor. | Non-drinkers: 0 drinks per week  Light drinkers: > 0 to < 3 drinks per week  Moderate drinkers: ≥ 3 to ≤ 7 drinks per week  Heavy drinkers: > 7 drinks per week  Binge drinking: > 4 drinks on one occasion |
| Holton et al.  2019 [21]  Ireland | Longitudinal prevalence of alcohol-medication interactions in older adults | N = 1,459  51% women  ≥ 65 years  Mean age 71.6 (±5.4) | Longitudinal, prospective study.  Data from three waves of the Irish Longitudinal Study on Ageing (2009-15). Community-dwelling older adults. In-home face-to-face computer-aided personal interview. | Self-reporting.  Assessed quantity and frequency of alcohol consumption with self-completion questionnaire. Calculated number of standard drinks consumed per week. One standard drink = 10 g of alcohol (= 1.25 UK alcohol units).  Recall period not reported. | Current drinkers:  Any alcohol consumption  Heavy drinkers:  Men: > 17 standard drinks/week  Women: > 11 standard drinks/week  Men/women: ≥ 6 standard drinks per drinking occasion |
| Iparraguirre et al.  2015 [22]  England | Socioeconomic determinants of risk of harmful alcohol drinking in adults | N = 9,275  53.5% women  ≥ 50 years  Mean age 66.6 (±9.0) | Longitudinal, prospective study.  Data from the English Longitudinal Survey of Ageing, wave 4 (2008-09) and wave 5 (2010-11). Community dwelling individuals. | Self-reporting.  Defined risk of harmful drinking following the guidelines set out by the National Institute for Health and Care Excellence.  1 glass of wine was equivalent to 2.1 units, 1 pint of beer to 2.8 units and 1 measure of spirits to 1 unit. Did not define alcohol content (g) in one unit.  Recall period not reported. | Lower risk drinking:  Men: ≤ 21 units/week  Women: ≤ 14 units/week  Increasing risk drinking:  Men: 22 ≤ 50 units/week  Women: 15 ≤ 35 units/week  Higher risk drinking:  Men: > 50 alcohol units/week  Women: > 35 units/week |
| Jeong et al.  2012 [23]  Korea | Association between alcohol consumption and mortality in older adults | N = 997  56.9% women  ≥ 65 years  Mean age 76.3 (±8.7)  Age range 65-98 | Longitudinal, prospective study.  Data from the Korean Longitudinal Study on Health and Aging (2005-06). Randomly sample. Community dwelling older adults.  Face-to-face clinical interviews. | Self-reporting.  Assessed alcohol consumption past 12 months. Assessed drinks per week, drinks per occasion and days per month. Used DSM-IV and AUDIT to define problematic drinking.  1 drink = 10 g of alcohol. | Lifetime abstainer: Never consumed alcohol  Ex-drinkers  Social drinkers: AUDIT score < 8  Light drinking: 0 < drinks ≤ 7 per week  Moderate drinking: 7 < drinks ≤ 14 per week  Heavy drinking: > 14 drinks/week  Binge drinking: ≥ 6 drinks on a single occasion at least monthly  Problematic drinking defined as at-risk drinking, alcohol abuse or alcohol dependence.  At-risk drinking: AUDIT score ≥ 8  Alcohol abuse or alcohol dependence: Defined according to DSM-IV |
| Liu et al.  2019 [24]  Japan | Association between alcohol consumption and dementia in older  adults | N = 53,311  65.8% women  Mean age: 71.4 (±7.5) | Longitudinal retrospective cohort study.  The Okayama Study. Followed from 2008 to 2014. The participants  completed a health checkup questionnaire from the Health Service of the municipal government. Community dwelling older adults. | Self-reporting.  Assessed currently alcohol consumption, the number of drinks per day, and drinking frequency (occasionally or daily). Recall period not reported.  A unit of alcohol defined as a beverage containing approx.  20 g alcohol. Equivalent to one bottle of beer, one glass of whiskey, 0.5 gou (a unit of drinks in Japan) of Japanese spirit. | Non-drinkers  Two units considered as the cut-off value between moderate and heavy alcohol consumption.  Moderate occasionally: ≤ 2 units/day  Moderate daily: ≤ 2 units/day  Heavy occasionally: > 2 units/day  Heavy daily: > 2 units/day |
| McCaul et al.  2010 [25]  Australia | Alcohol consumption and mortality in older adults | Men n = 11,727  ≥ 65 years  Age range 65-83  Women n = 12,432  ≥ 70 years  Age range 70-75 | Longitudinal, prospective study.  Data from the Health in Men Study and the Australian Longitudinal Study of Women’s Health (baseline data 1996). Population based cohort studies. Community-dwelling older adults. | Self-reporting.  Assessed frequency of use (number of days alcohol was consumed per week) and quantity consumed per day.  1 standard drink = 10 g of alcohol.  Recall period not reported. | Rarely drinking: Reported alcohol consumption, but not in the past year  Alcohol consumption < 1 time a week  Frequency:  1-2, 3-4, 5-6, 7 days/week  Quantity:  1-2, 3-4, 5-8, 9+ drinks/day |
| McEvoy et al.  2013 [26]  USA | Changes in alcohol consumption in older adults | N = 1,076  57% women  ≥ 50 years  Mean age 66.4  Age range 50-89 | Longitudinal, prospective study.  Data from the Rancho Bernardo cohort (1984-87 and 2007-09)  Community-dwelling older adults. | Self-reporting.  Assessed ever use of alcohol, alcohol consumption in the past year, and the frequency of alcohol consumption.  Assessed number of drinks consumed during an average week. Calculated the average weekly alcohol consumption.  1 standard drink = 12 g of alcohol.  Recall period not reported. | Consistent no-drinkers: Reported no past-year alcohol use at each visit.  Consistent drinkers: Reported some past-year alcohol use at each visit.  Inconsistent drinkers: Reported past-year alcohol use at some visits, but not at others.  Quitters: Stopped drinking at some points during the follow-up.  Weekly alcohol intake  Near daily alcohol intake  Mean weekly alcohol consumption: g/week  Drinking in excess of NIAAA guidelines: > 1 drink/day for women of any age and men ≥ 65 years, > 2 drinks/day for men < 65 years. |
| Merrick et al.  2011 [27]  USA | Association between alcohol consumption and hospitalization in older adults | N = 5,570  44.8% women  ≥ 65 years | Longitudinal study.  Data from the Medicare Current Beneficiary Survey (2001-06). Nationally representative sample. Community dwelling older adults. Past years drinkers. In-person, computer-assisted interviews. | Self-reporting.  Asked quantity-frequency questions. Recall period 12 months. Assessed heavy episodic drinking last year. Categorized drinking patterns according to NIAAA guidelines for older adults.  Did not define alcohol content (g) in one drink. | Within-guidelines drinkers:  ≤ 30 drinks/month, or ≤ 3 drinks on a single day.  Exceeding the monthly limit, but not single day limit:  > 30 drinks/months, ≤ 3 drinks on a single day.  Heavy episodic drinking: ≥ 4 drinks in a single day in a typical month last year. |
| Ormstad et al.  2016 [28]  Norway | Association between alcohol consumption and dementia related death | N = 25,635  46.4% women  ≥ 60 years  Mean age 69.8  Age range 60-80 | Longitudinal, prospective study.  Data from the Cohort of Norway (CONOR) (1994). Study setting, and participants not clearly described | Self-reporting.  Assessed frequency of alcohol consumption.  Recall period not reported. | Abstainer  Several times a week  Once per week  2-3 times a month  About once or less per month |
| Ortola et al.  2017 [29]    Spain | Association between alcohol consumption and falls in older adults | N = 2,170  53.4% women  ≥ 60 years  Mean age 68.7 (± 6.4) | Longitudinal, prospective study.  Data from the Seniors-ENRICA cohort (2008-12). Community-dwelling older adults.  In-home interviews. | Self-reporting.  Assessed the usual alcohol consumption in the previous year with a validated diet history. Collected information on 34 alcoholic beverages and used photographs to help quantify portion sizes. Used standard beverage composition table to estimate alcohol content.  Assessed binge drinking in the preceding 30 days. | Never drinkers: Including occasional drinkers with average alcohol intake close to zero.  Ex-drinkers  Moderate drinkers:  Men: < 40 g/day  Women: < 24 g/day  Heavy drinkers:  Men: ≥ 40 g/day  Women: ≥ 24 g/day  Mediterranean drinking pattern:  < 40 g/day for men, < 24 g/day for women, no binge drinking, with preference for wine and drinking only with meals.  Binge drinking: ≥ 80 g in men,  ≥ 60 g in women, during any drinking session in the preceding 30 days. |
| Ortolá et al. 2019 [30]  Spain | Association between changes in health status and changes in alcohol consumption in older adults | N = 571  Women 38.7%  ≥ 60 years  Mean age: 70.1 (±5.2) | Longitudinal study.  Data from the Seniors-ENRICA cohort (2008–2010). Followed-up for 8.2 years. Four waves of data collection (2008, 2012, 2015 and 2017). Community-dwelling adults. Computer-assisted telephone interview and home visits. | Self-reporting.  Assessed usual alcohol consumption last 12 months. Used a validated diet history. Collected information on 34 alcoholic beverages. Used photographs to better quantify portion sizes. Alcohol content of each beverage was estimated using standard composition tables. | Never drinkers: average alcohol intake of 0 g/day  Ex-drinkers: average alcohol intake of 0 g/day who answered, ‘I used to drink alcohol, but I quit’. Current drinkers: average alcohol intake > 0 g/day |
| Richard et al.  2017 [31]  USA | Association between alcohol consumption and cognitive function in older adults | N = 1,344  54.2% women  Mean age 67.4-72.1  (± 6.1-6.5) | Longitudinal, prospective study.  Data from the Rancho Bernardo Study (1984-2009). Community dwelling older adults. | Self-reporting.  Assessed the amount and frequency of alcohol consumption. Assessed alcohol consumption with a self-administered standardized questionnaire. Asked about ever drunk alcohol, if they had drunk alcohol past 12 months, drinking frequency, and how many drinks they consumed during an average week. Calculated the average weekly alcohol intake.  1 standard drink = 12 g of ethanol. | Non-drinkers: Life-time abstainers and those who did not drink within the last year (former drinkers)  Moderate drinkers:  Men ≥ 65 years and women:  ≤ 1 drink/day  Men < 65 years: ≤ 2 drinks/day  Heavy drinkers: Men ≥ 65 years and women: > 1-3 drinks/day  Men < 65 years: > 2-4 drinks/day  Excessive drinkers: > 3 drinks/day for men ≥ 65 years and women, > 4 drinks/day for men < 65 years  Frequency of alcohol consumption:  Non-drinkers  Infrequent drinkers: < 2 times/month  Weekly: 1-4 times/week  Near daily drinkers: 5-7 times/week |
| Scott et al.  2020 [32]  USA | Association between moderate alcohol use and mood and  functional ability in later life | ≥ 65 years  Model 1: mood:  N = 2,294  65.4% women  Mean age: 76 (± 7)  Model 2: functional ability:  N = 2,357  65% women  Mean age: 76 (± 7) | Study 1: cross-sectional.  Study 2: longitudinal.  Data from the Health and Retirement Study (2012-214). Prospective  cohort study. Study setting, and participants not clearly described  In-person and telephone interviews. | Self-reporting.  Asked how many days per week the participants had any drink containing liquor. Assessed how many drinks consumed on drinking days. Recall period 3 months. Did not define alcohol content (g) in one drink. | Abstainer: denied drinking alcohol  Moderate drinkers:  ≤ 3 drinks/day and ≤ 7 drinks/week  Excluded participants who reported alcohol use above moderate alcohol consumption criteria. |
| Shaw et al.  2011 [33]  USA | Association between financial strain and alcohol consumption in older adults. | N = 2,352  60% women  ≥ 65 years  Mean age 76.5 (± 8.2) | Longitudinal, prospective study.  Data from six waves of a nationwide longitudinal survey of older adults (1992-2006). Randomly selected national sample. Community dwelling older adults. | Self-reporting.  Assessed ever use of alcohol, drinking frequency last month, and number of drinks consumed on days they drank. Calculated the quantity of alcoholic drinks consumed per month by multiplying the number of drinking days a month by the number of drinks consumed per day. Defined drinking patterns according to NIAAA recommendations for older adults. Did not define alcohol content (g) in one drink. | Heavy drinking:  > 30 drinks/months |
| Shiotsuki et al.  2019 [34]  Japan | Association between alcohol consumption and ischemic stroke | N = 60,836 cases of strokes  Women 46%  Median age women: 76 (60-76)  Median age men: 68 (66-83) | Longitudinal retrospective study.  Data from the Japan Standard Stroke Registry (2013). Medical information assessed at hospital admission and discharge. | Self-reporting.  Assessed the amount of alcohol intake prior to stroke onset.  Did not define alcohol content (g) in one drink.  Recall period not reported. | 0 = Nondrinker  1 = Occasional drinker  2 = Current drinker: 20-39 g/day  3 = Current drinker: 40-59 g/day  4 = Current drinker: ≥ 60 g/day  5 = Past heavy drinker: defined as a habitual drinker who was advised by a physician to stop drinking at least 1 year before stroke onset. |
| Siddiquee et al.  2020 [35]  Japan | Association between alcohol consumption and cognitive function in elderly men | N = 585  100% men  ≥ 65 years  Mean age: 70.3-73.6 (± 3.8-4.4). | Longitudinal study.  Data from the Shiga Epidemiological Study of Subclinical Atherosclerosis (SESSA: 2006-2008). Prospective, population-based study. Follow up: 2012-2015. Self-reported questionnaire.  Community dwelling older men. | Self-reporting.  Current drinkers were asked how frequently they drank alcohol in a typical week or month. Assessed type (i.e., wine and beer), number, and portion size of alcohol consumption. Calculated grams of alcohol per week using following conversion equation: Ethanol (g/week) = number of days/week consumed x number consumed x portion size x concentration x density of ethanol to water (constant) + other ethanol. | Never drinkers: Never drunk alcohol.  Ex drinkers: quit before interview  Current drinkers: Drunk alcohol in the past week or month  Very light drinkers: < 14 g/day  Light drinkers: 14-23 g/day  Moderate drinkers: > 23-46 g/day  Heavy drinkers: > 46 g/day  Considered “one drink” (14 g of ethanol) (Western standard), and “gou” (23 g of ethanol), a  “standard” drink in Japan, when making these drinking groups. |
| Soler-Vila et al.  2019 [36]  Spain | Associated factors with changes in alcohol consumption among older adults | N = 2,505  Women 53.3%  > 60 years  Mean age: 68.7 | Longitudinal study.  Data from Seniors-ENRICA. A prospective population-based cohort study (2008-2010).  Stratified cluster random sampling. Representative non-institutionalized population. Computer-assisted telephone interviews and structured questionnaires. Two home visits to assess diet and alcohol history. | Self-reported.  Used a validated diet history to assess alcohol consumption at baseline (2008–10) and at follow-up (2012). Collected detailed information on habitual average alcohol consumption in the last 12 months and binge drinking in the last 30 days.  1 US standard drink = 14 g alcohol | Differentiated between non-drinkers and ex drinkers.  Ex drinkers: quit drinking and reported 0 g/day of alcohol intake last 12 months.  Non-drinkers: life-long abstainers and very occasional drinkers (individuals who reported 0 g/day  of alcohol intake in the last year, but self-described as drinkers).  Light drinkers: ≤ 1 standard drink/day and no binge drinking in the last 30 days.  At risk drinkers: > 1 standard drink (>14 g of alcohol) per day or any binge drinking in the last 30 days (≥ 80 g for men and ≥ 60 g for women of alcohol in one session).  Defined three patterns of drinking change over time:  1. Light drinker to At risk drinker  2. Light drinker to Ex drinker  3. At risk drinker to Light drinker |
| Tait et al.  2013 [37]  Australia | Association between alcohol consumption, hospital admissions, and falls in older adults | N = 16,785  85.5% women  ≥ 65 years at baseline | Longitudinal,  retrospective study.  Data from five cohort studies (1990-2002):  The Dynamic  Analyses to Optimise  Ageing (DYNOPTA) cohort. Population based.  Community-dwelling older adults. | Self-reporting.  Assessed average quantity and frequency of alcohol use. Alcohol consumption categorized according to Australian guidelines.  1 standard drink = 10 g of alcohol.  Recall period not reported. | Abstainers  Low risk: > 0 ≤ 2 drinks/day  Long term risk: > 2 ≤ 4 drinks/day  Short-term risk: > 4 drinks/day |
| Tateishi et al.  2019 [38]  Japan | Association between alcohol consumption and non-B, non-C hepatocellular carcinoma (HCC) | N = 2,087 patients with HCC  25.3% women  Median age: 73 (66-78) | Longitudinal, retrospective study.  A nationwide survey.  Included patients diagnosed with non-B, non-C hepatocellular carcinoma at the participating hospital (2011-2015). Collected data at the time of the initial diagnosis of HCC. Thirty-four hospitals participated. | Self-reporting.  Assessed daily alcohol consumption.  Daily alcohol consumption was calculated from alcohol intake and frequency.  Did not define alcohol content (g) in one drink.  Recall period not reported. | Daily alcohol consumption:  20 g/day  21-59 g/day  ≥ 60 g/day |
| Tevik et al.  2019 [39]  Norway | Morality in older adults with frequent alcohol consumption and use of drugs with addiction potential | N = 11,545  52.7% women  ≥ 65 years  Mean age 73.7 (± 6.3) | Longitudinal, prospective study.  Data from The Nord-Trøndelag Health Study (HUNT3, 2006-08). Population based study.  Community-dwelling older adults. | Self-reporting.  Assessed drinking frequency last 12 months. | Never drinkers  Non-drinkers last year: Not consumed alcohol last year  Occasional drinkers: Alcohol consumption a few times a year  Current drinkers: At least alcohol consumption a few times a year  Frequent drinkers: ≥ 4 days/week |
| van Oort et al.  2020 [40]  The Netherlands | Association between moderate and heavy alcohol consumption and left ventricular ejection fraction | Cross sectional analyses:  N = 778  49% women  Mean age: 68.4 (± 7.2)  Prospective analyses:  N = 404  48% women  Mean age: 66.6 (± 6.3) | Longitudinal study.  Data from the Hoorn Study (2000-2001). Population-based prospective cohort study. Eight years follow up: 2007-2009.  Community dwelling older adults. | Self-reporting.  Assessed alcohol consumption with a validated food-frequency questionnaire. Recall period last year.  Assessed frequency and types of consumed alcoholic beverages (beer, wine, liquor). Calculated the total weekly alcohol consumption by adding up the reported weekly frequencies of all alcoholic beverages.  Calculated grams of alcohol per week. One standard alcoholic beverage = 10 g alcohol. | None: 0 g/week  Light drinkers: > 0 – 30 g/week Light-to-moderate drinkers:  > 30 - ≤ 70 g/week  Moderate drinkers: > 70 - ≤ 140 g/week  Heavy drinkers: >140 g/week |
| Villalonga-Olives et al. [41]  2020  USA | Association between perceived social capital and binge drinking in older adults | N = 19,140  Included both women and men.  Mean age: 66.8 (±10.3) | Longitudinal study.  Data from the Health and Retirement Study (2006–2014). Nationally representative sample. Probability sample of households. Community dwelling older adults. | Self-reporting.  Assessed binge drinking last three months. Asked how many days the participants had ≥ 4 drinks on one occasion. Response alternative from 0 to 92 days. Did not define alcohol content (g) in one drink. | Binge drinking:  ≥ 4 drinks on one occasion |
| Weyerer et al.  2011 [42]  Germany | Association between alcohol consumption and dementia in older adults | N = 3,202  37.6% women  ≥ 75 years  Mean age 80.2 | Longitudinal, prospective study.  Data from individuals attending general practitioners in Germany (years not reported). Interviewed in their homes. Community dwelling older adults. | Self-reporting.  Assessed frequency of alcohol consumption and quantity of beer, wine, and liquor. Converted alcohol intake to grams of alcohol per day. 1 standard drink = 10 g of alcohol. | Quantity of alcohol consumption:  Abstinent  1-9, 10-19, 20-29, 30-39, ≥ 40 g alcohol/day  Type of alcohol:  Abstinent  Wine only  Beer only  Mixed (wine, beer, and other alcoholic beverages)  Harmful drinking:  Men: > 60 g alcohol/day  Women: > 40 g alcohol/day  Extremely high consumption:  Men: > 120 g alcohol/day  Women: > 80 g alcohol/day |
| **Author**  **Year**  **Country** | **Aim of the study** | **Participants: numbers, gender, age** | **Cross-sectional studies**  **Data**  **Sample** | **Method** | **Definition and measure** |
| Aalto et al.  2011 [43]  Finland | Validity of AUDIT in screening for heavy drinking in older adults | N = 517  49.7% women  ≥ 65 years  Mean age 69 (± 2.8)  Age range 65-74 | Cross-sectional study.  Data from The National FINRISK study (2007).  Stratified random sub-sample. Community dwelling older adults.  Interview regarding alcohol consumption.  Self-reporting regarding AUDIT questionnaire. | Self-reporting.  Timeline follow-back interview regarding alcohol consumption over a period of time (28 days) prior to the interview.  Assessed alcohol consumption with AUDIT (10 questions) and AUDIT-C (3 questions). Alcohol volume was converted into Finnish standard drinks (33 cl bottle of beer, 12 cl glass of wine, 4 cl drink of spirits).  1 standard drink = 12 g of alcohol. | Heavy drinking: ≥ 8 drinks (≥ 96 g alcohol) in a week or ≥ 4 drinks (≥ 48 g alcohol) at least in 1 day last 28 days  Heavy drinking: Optimal cut-point of the AUDIT  ≥ 5 (sensitivity 0.86 and specificity 0.87)  Heavy drinking: Optimal cut-point of the AUDIT-C  ≥ 4 (sensitivity 0.94 and specificity 0.80) |
| Ahlner et al.  2018 [44]  Sweden | Compared alcohol consumption patterns among birth cohorts of older adults born 1906-1907, 1922, 1930 and 1944 | N = 2,268  Included both women and men  ≥ 70 years | Cross-sectional study.  Used a serial cross-sectional design.  Data from four waves of the Gothenburg H70 Birth Cohort Studies (1976-77, 1992-93, 2000-02 and 2014-16). Individuals living in both private households and residential care. Serial cross-sectional design. Face to face interviews. | Self-reporting.  Assessed drinking frequency last month for each beverage type. Assessed weekly quantity of alcohol consumption (beer, wine, and spirits) last month. Estimated grams of weekly alcohol consumption using conversion factors (spirits 1 cl = 3 g, wine 1 cl = 1 g, beer > 3.5% 1 cl = 1/3 g). | Drinking frequency:  Never, ≤ 2 days/week, 3-5 days week, > 5 days/week  Weekly alcohol consumption:  0, 1-20, 20-40, 40-60, 100-150, 150-250, 250-500 or > 500 g alcohol/week  At risk alcohol consumption:  ≥ 100 g alcohol/week |
| Barnes et al.  2010 [45]  USA | Prevalence of at risk-drinking among older adults | N = 3,308  48.2% women  ≥ 60 years  Included only current drinkers defined as consumed at least one alcoholic drink in the past three months. | Cross-sectional study.  Data from Project Senior Health and Alcohol Risk Education (year not reported). Included participants accessing primary care clinics.  Mailed survey. | Self-reporting.  Assessed alcohol consumption by the tool CARET. Recall period 12 months. | At risk drinking from CARET based on  - alcohol use behaviors (e.g., quantity and frequency of alcohol)  - combination of quantity and frequency of alcohol use with selected comorbidities or  - combination of quantity and frequency with medication (e.g., antidepressants, sedatives) |
| Bryant et al.  2013 [46]  Asian and Hispanic immigrants in USA | Association between acculturation and alcohol consumption patterns among older immigrants | N = 1,829  ≥ 60 years  Asian n = 1,264  56.6% women  Mean age: 69.6 (±7.1)  Hispanic n = 571  63.1% women  Mean age: 68.4 (±7.0) | Cross-sectional study.  Data from the California Health Interview Survey (2009). Random sample Community dwelling adults. Telephone survey. | Self-reporting.  Assessed past year alcohol consumption, quantity, and frequency of alcohol consumption and binge drinking. Did not define alcohol content (g) in one drink. | Past year alcohol consumption:  Consumed any alcohol use in the past 12 months.  Past year binge drinking:  Men: ≥ 5 drinks in one day  Women: ≥ 4 drinks in one day  Number of binge-drinking days:  Range 0-365 days |
| Bryant et al.  2013 [47]  USA | Association between binge drinking and psychological distress among older adult drinkers | N = 13,265  57.5% women  ≥ 60 years who had consumed alcohol past year.  Mean age: 71.1 (±7.9) | Cross-sectional study. Data from the California Health Interview Survey (2009). Random sample. Community dwelling adults. Telephone survey. | Self-reporting.  Assessed frequency of binge drinking in the past year.  Did not define alcohol content (g) in one drink. | Binge drinking:  Men: ≥ 5 drinks in one sitting  Women: ≥ 4 drinks in one sitting  Frequency of binge drinking:  From none past year to daily |
| Bryant et al.  2019 [48]  USA | Association between regional and racial/ethnic variation and alcohol consumption in older adults | N = 185,190  64.4% women  ≥ 60 years  Mean age: 71.4 (± 8.3) | Cross-sectional study.  Data from the Behavioral Risk Factor Surveillance System  (2010).  Community dwelling older adults.  Data collected by telephone interview. | Self-reporting.  Assessed number of days alcohol was consumed in the past month and number of drinks consumed per drinking day in the past month.  One drink was equivalent to a 12-ounce beer, a 5-ounce glass of wine, or a drink with one shot of liquor. | Calculated mean monthly drinks |
| Chan et al.  2010 [49]  China | Association between alcohol consumption and cognitive impairment in older adults | N = 314  52.2% women  ≥ 65 years  Mean age: 79.9 (±6.5) | Cross-sectional study.  Data from the Geriatric Clinics of Queen Mary Hospital and Grantham Hospital of Hong Kong (2008). Participants hospitalized patients.  Face-to-face interview. | Self-reporting.  Assessed the volume of alcohol beverages which were converted into grams of weekly pure ethanol from alcohol units.  1 unit = 8 g of alcohol.  The average weekly alcohol consumption was calculated by multiplying the frequency of consumption in a typical week by the amount of ethanol in the consumed beverage. Recall period not reported. | Ex-drinkers:  Drank in the past, but stopped drinking currently  Non-drinkers: 0 g/week  Light drinkers:  Men: < 168 g/week  Women: < 112 g/week  Moderate drinkers:  Men: > 168 g, but < 400 g/week  Women: > 112 g, but < 280 g/week  Heavy drinkers:  Men: > 400 g/week  Women: > 280 g/week |
| Choi et al.  2011 [50]  USA | Association between alcohol consumption and depressive symptoms in older adults | N = 2,094  51.8% women  ≥ 57 years  Mean age: 68.1 (±7.7)  Age range 57-85 | Cross-sectional study.  Data from the National Social Life, Health and Aging Project (2005-06).  Nationally, representative sample. Community-dwelling adults. Face-to-face interview. | Self-reporting.  Drinking frequency:  Assessed average number of drinking days per week in the preceding 3 months.  Quantity/amount:  Assessed by the average number of drinks the respondent had had on the days they drank. Multiplied the average number of drinking days per week by the average number of drinks per drinking day. Did not define alcohol content (g) in one drink. | Number of drinking days per week:  0 days (non-drinkers)  1-7 days  Number of drinks on drinking days:  Men: 0-4 drinks/day  Women: 0-3 drinks/day  Number of total drinks per week:  Mean/median drinks  Heavy/binge drinking:  Men: ≥ 4 drinks per drinking day  Women: ≥ 3 drinks per drinking day |
| Cousins et al.  2014 [51]  Ireland | Prevalence of potential alcohol and drug interactions in older adults | N = 3,815  53.4% women  ≥ 60 years  Mean age 69.7 (± 7.3) | Cross-sectional study.  Data from the Irish Longitudinal Study on Aging (2009-11). Nationally representative population. Community dwelling older adults. Face-to-face interview in the participant’s home. | Self-reporting.  Assessed current alcohol consumption last 6 months. Calculated number of drinks consumed per day/per week using self-reported quantity and frequency measures (standard quantity-frequency approach). Alcohol consumption patterns categorized according to NIAAA age specific guidelines. The limit was set based on standard drinks in Ireland: 1 standard drink = 10 g alcohol. Problem drinking assessed by CAGE. | Non-drinkers  Current drinkers: Drinking alcohol in the previous 6 months  Light/moderate drinkers:  ≤ 10 drinks/weeks  ≤ 4 drinks/day  Heavy drinkers:  In excess of NIAAA limits  > 10 drinks/week  > 4 drinks/day  Problem drinking: CAGE score ≥ 2 |
| D’Ovidio et al.  2019 [52]  Ireland, The Netherlands, and Italy | Association between alcohol consumption and amyotrophic lateral sclerosis (ALS) | Cases: n = 1,557 patients with ALS.  40-47% women  Median age: 65-67  Controls: n = 2,922  40-47% women  Median age: 65-68 | Case-control study with cross sectional design.  Data from the Euro-MOTOR study. Clinical  data collected from each ALS Centre through patients’ medical records. Population-based design. Paper- and pencil survey. | Self-reporting.  Assessed alcohol consumption using the following indicators: ever exposed to alcohol; years of start and cessation of alcohol consumption; number of years of abstinence; number of glasses of alcohol per week. Distinguished between consumption of general alcoholic beverages and red wine.  Did not define alcohol content (g) in one drink. | Ever drunk alcohol  Ever drunk red wine  Drinking alcohol status:  - Never  - Current  - Former  Drinking red wine status  - Never  - Current  - Former  Lifetime cumulative dose Expressed in quartiles of lifetime total units of alcoholic beverages. |
| Davis et al.  2014 [53]  Iceland | Association between alcohol consumption and cognitive function in older adults | N = 3,363  59.1% women  Mean age range in women: 74-76.8  Mean age range in men: 75-77 | Cross-sectional study.  Data from the Age Gene/Environment Susceptibility-Reykjavik Study (2002-2006).  Random sample. Study setting, and participants not clearly described. | Self-reporting.  Assessed drinking frequency last month, and how many drinks consumed on a single occasion. Multiplied number of occasions by amount consumed on each occasion to calculate the number of drinks consumed per month. Multiplied the product by 14 g of ethanol per drink and divided by 3.25 to determine g of ethanol per week. | **Men:**  Very light drinkers: < 1 drink/week  Light drinkers: 1-7 drinks/week  Moderate drinkers: 7-14 drinks/week  Heavy: > 14 drinks/week  **Women:**  Very light drinkers: < 1 drink/week  Light-to-moderate: 1-7 drinks/week  Heavy: > 7 drinks/week  **Women/men**  Heavy drinking/binge drinking:  ≥ 5 drinks on any occasion within the past 30 days |
| Forlani et al.  2014 [54]  Italy | Association between anxiety and alcohol consumption in older adults | N = 366  50.3% women  ≥ 74 years  Mean age 83.7 (±6.2)  Age range 74-79 | Cross-sectional study.  Data from a study on affective and cognitive effects of ageing (2006). Community-dwelling individuals. Face-to-face interview. | Self-reporting.  The quantity of each beverage drunk was converted into unit/day. One unit = one glass of 125 ml wine, a can of 330 ml beer, and a small glass of 40 ml hard liquor. Did not define alcohol content (g) in one unit. | Four group of current alcohol consumption:  < 1 alcohol unit/day  1 alcohol unit/day  2 alcohol units/day  > 2 alcohol units/day |
| Foster et al.  2019 [55]  England | Prevalence of simultaneous use of  alcohol and prescription medication in  older adults | N = 2,169  52.2% women  ≥ 65 years  Mean age: 73.7 (± 6.5)  Age range: 65–89 | Cross-sectional study. Data from the Health Survey of England (2013). General population survey. Stratified random probability sample of private households. Community dwelling older adults. | Self-reporting.  Assessed alcohol consumption with the use of a drinking diary concerning the amount of alcohol consumed in the last 7 days. The amount was converted into units of alcohol consumed per week.  1 unit = 8 g alcohol.  One unit is equivalent to half a pint of normal strength beer. | Alcohol consumption last 7 days: None (last 12 months); < 1 unit; 1–7 units; > 7–10 units; > 10–14 units; > 14 – 21 units; > 21–28 units; > 28–35 units; > 35–50 units; > 50 units  Alcohol consumption last 7 days: None or < 1 unit last 12 months; 1-10 units; > 10-21 units; > 21-28 units; > 28-50 units; > 50 units.  Definition 1: At risk drinking:  >10 units of alcohol per week (according to Royal College of Physicians).  Definition 2: At risk drinking:  > 14 units of alcohol per week (according to the Department of Health at risk drinking guidelines). |
| Fuentes et al.  2017 [56]  16 European countries (South, Central, North, and West) | Prevalence of binge drinking in European older adults | N = 58,489  57% women  ≥ 50 years  Mean age women:  67.4 years  Mean age men:  65.4 years | Cross-sectional study.  Data from the Survey of Health, Ageing and Retirement in Europe (2011-12).  Probabilistic sample. Community-dwelling adults. | Self-reporting.  Assessed current alcohol consumption, frequency, quantity, and binge drinking. Recall period 3 months. Current drinkers were categorized as binge drinkers or no-binge drinkers. Did not define alcohol content (g) in one drink. | Never  Former  No-binge drinkers  Binge drinkers:  ≥ 6 standard drinks on one occasion at least monthly |
| Gibson et al.  2017 [57]  Jamaica | Alcohol use in older adults | N = 2,943  52% women  ≥ 60 years  Mean age 72.2 (± 8.9)  Age range 60-103 | Cross-sectional study.  Data from a nationally representative health and lifestyle survey (2012). Community dwelling older adults. | Self-reporting.  Assessed current alcohol use. Recall period 12 months. | Current alcohol use: Having drunk alcohol during the last 12 months. |
| González-Rubio et al.  2016 [58]    Spain | Association between alcohol consumption and health behavior and quality of life in older adults | N = 231  48-59% women  ≥ 55 years  Mean age range 67.3-68.8  Age range 55-85 | Cross-sectional study.  Data from an observational study (2012-13). Community dwelling older adults.  Included participants with moderate alcohol consumption. | Self-report.  Interviewed by a trained nutritionist with a recall questionnaire specific for alcoholic drinks consumed in the last year. Assessed frequency and volume. Total grams of alcohol intake per day were calculated according to alcohol content per 100 ml of each alcoholic beverage (wine 11 g, beer 4.5 g, cider 5 g, liquors 20 g).  1 standard drink = 10 g of alcohol. | Abstainers  Occasional drinkers:  < 4 drinks/month  Moderate drinkers:  Men: < 40 g alcohol/day  Women: < 25 g alcohol/day |
| Guidolin et al.  2016 [59]  Brazil | Patterns of alcohol consumption in older adults | N = 557  63.7% women  ≥ 60 years  Mean age 69 (±7.1)  Age range 60-103 | Cross-sectional study.  Data from a Family Health Strategy program (year not reported)  Community residents and hospitalized participants.  In home-interview and in-hospital interview. | Self-reporting.  Data related to alcohol abuse and alcoholism obtained via the Mini International Neuropsychiatric Interview. Interviewed by trained physicians. Recall period 12 months. | Current alcohol dependence:  ≥ 3 of 7 criteria last 12 months  Current alcohol abuse:  ≥ 1 of 4 criteria last 12 months  Previous alcohol dependence: Same criteria for alcohol dependence, but a year ago on abstinence.  History of alcoholism |
| Hajek et al.  2017 [60]  Germany | Factors associated with alcohol consumption in adults | N = 7,820  50.9% women  ≥ 40 years  Mean age 65.5  Age range 40-95 years | Cross-sectional study.  Data from the nationwide German Aging Study (2014). Population-based sample. Community dwelling adults.  Face-to-face computer-assisted interview at participants homes and drop-off questionnaire. | Self-reporting.  Assessed alcohol frequency. Recall period not reported. | Never drinkers  Occasional drinkers: Rarer than once a month, one to three times a month, once a week, or several times a week  Non-risky alcohol intake: No daily alcohol consumption  Risky alcohol intake: Daily alcohol consumption |
| Han et al.  2019 [61]  USA | Prevalence of binge drinking among older adults | N = 10,927  55% women  ≥ 65 years | Cross-sectional study.  Data from the US National Survey on Drug Use and Health.  (2015-2017). Noninstitutionalized  individuals. Nationally  representative probability sample. Computer-assisted interviewing (conducted by an interviewer) and audio computer-assisted self-interviewing. | Self-reporting.  Assessed alcohol use and binge drinking in the past month. The definition based on the NIAAA’s definition.  Did not define alcohol content (g) in one drink. | Binge drinking:  Men: ≥ 5 drinks on the same occasion.  Women: ≥ 4 drinks on the same occasion. |
| Heegaard et al.  2011 [62]  Denmark | Association between type of alcohol consumption and missing teeth in older adults | N = 783  Included both women and men  ≥ 65 years  Age-range 65-95 | Cross-sectional study.  Data from the Copenhagen City Heart Study (2004-05). Population-based study. Community-dwelling older adults. Face-to-face interview. | Self-reporting.  Assessed total weekly alcohol consumption, beverage-specific alcohol consumption, beverage preference (defined as the highest intake of one beverage type).  One beverage = 12 g of alcohol.  Recall period last week. | Abstainers: < 1 beverage/week  Moderate drinkers:  Men: 1-21 beverages/week  Women: 1-14 beverages/week  Heavy drinkers:  Men: > 21 beverages/week  Women: > 14 beverages/week  Type-specific weekly intake (wine, beer, and spirits):  0, > 0-6, > 6 drinks/week |
| Hoeck et al.  2013 [63]  Belgium | Patterns of alcohol consumption in older adults | N = 3,954  56.7% women  ≥ 65 years  Mean age 74.9 (±0.1) | Cross-sectional study.  Data from the Belgian Health Interview Survey (2001-04).  Community dwelling older adults. | Self-reporting.  Assessed weekly average of consumed glasses of alcohol. Did not define alcohol content (g) in one glass of alcohol.  Alcohol problems measured with the CAGE.  Recall period last week. | Non-drinkers or occasional drinkers: Consumed a mean of 0 glasses alcohol/week  Moderate drinkers: 1-7 glasses/week  At-risk drinkers: 8-14 glasses/week  Heavy drinkers: 15-21 glasses/week  Problematic drinkers: > 21 glasses/week  Alcohol problem: CAGE score ≥ 2 or CAGE 65+ score ≥ 1 |
| Hongthong et al.  2016 [64]  Thailand | Factors predictive of alcohol consumption in older adults | N = 400  62% women  ≥ 60 years  Mean age 71.9 (±8)  Age range 60-97 | Cross-sectional study.  Data from the survey on Quality of Life among Thai older people (2014). Random sampling. Community dwelling older adults.  Face-to-face interview. | Assessed drinking pattern and drinking frequency in their lifetime.  Recall period not reported. | Never consumed  Current drinkers: consumed alcohol  Moderate drinkers: 0.5-1 standard drink per drinking episode  Drinking frequency among current drinkers:  Less than once a month  1-3 times/month  1-5 days/week  5-6 days/week |
| Ilomaki et al.  2013 [65]  Australia | Association between psychotropic drug use and alcohol consumption in older men | N = 1,705  100% men  ≥ 70 years | Cross-sectional study.  Data from the Concord Health and Ageing in Men Project (2005-07).  Random sample. Community-dwelling older men. | Self-reporting.  Assessed alcohol consumption during the past 12 months. Calculated the average number of drinking days per week, month or year, the average number of drinks per drinking day, and the number of days when ≥ 5 drinks were consumed. Computed a quantity-frequency measure. Assessed problematic drinking with CAGE.  1 standard drink = 10 g of alcohol. | Non-drinkers: Did not consume any alcohol during the previous 12 months. Non-drinkers further categorized as never drinkers (had not consumed > 12 alcoholic drinks during their lifetime) or former drinkers.  Daily drinkers: Alcohol on 7 days per week  Binge drinkers: ≥ 5 drinks at least once per month  Heavy drinkers: > 2 drinks/day  Problem drinkers: CAGE score ≥ 2 |
| Ilomaki et al.  2014 [66]  Australia | Prevalence of alcohol consumption in older men | N = 1,705  100% men  ≥ 70 years | Cross-sectional study.  Data from the Concord Health and Ageing in Men Project (2005-07).  Random sample of community-dwelling older men. | Self-reporting.  Assessed alcohol consumption during the past 12 months. Calculated the average number of drinking days per week, month, or year; the average number of drinks per drinking day; and the number of days when ≥ 5 drinks were consumed. Computed a quantity-frequency measure.  Assessed problematic drinking with CAGE.  1 standard drink = 10 g of alcohol. | Non-drinkers: < 12 drinks during the previous 12 months. Non-drinkers further categorized as never drinkers (had not consumed > 12 drinks during their lifetime), or former drinkers.  Current drinkers: ≥ 12 drinks during the previous 12 months  Moderate drinking: ≤ 2 drinks/day  Heavy drinking: 3-4 drinks/day  Excessive drinking: > 4 drinks/day  Binge drinking: ≥ 5 drinks on a single day at least once per month  Daily drinking: Alcohol on 7 days/week  Problem drinking: CAGE score ≥ 2 |
| Immonen et al.  2011 [67]  Finland | Prevalence of at-risk drinking in older adults | N = 1,395  62.7% women  ≥ 65 years  Mean age 78 | Cross-sectional study.  A postal questionnaire to a random sample from the Espoo Population Register (2007). Community dwelling older adults. | Self-reporting.  Assessed number of drinks on a typical drinking day (quantity) and frequency of alcohol consumption. Asked how often they had ≥ 3 drinks on one occasion.  1 standard drink = 12 g of alcohol.  Recall period not reported. | Frequency of alcohol consumption:  < once a month or not at all  > once a month but < 3 times/week  > 3 times/week  At-risk drinking: Consuming > 7 drinks/week or ≥ 5 drinks on a typical drinking day or using ≥ 3 drinks several times per week. |
| Immonen et al.  2013 [68]  Finland | Prevalence of potential alcohol-drug interactions in older adults | N = 2,100  64.5% women  ≥ 65 years  Mean age 78.1 | Cross-sectional study. Data from the Espoo Population Register (2007). Stratified random sample. Community dwelling older adults.  Postal questionnaire. | Self-reporting.  Assessed alcohol consumption with questions from AUDIT. Assessed drinking frequency and quantity (how many drinks in a typical day). Assessed how often they consumed ≥ 3 drinks on one occasion.  1 standard drink = 12 g of alcohol.  Recall period not reported. | Minimal/non-users: < 1 drink/month  Moderate users: ≥ 1 drink/month, but ≤ 7 drinks/week  At-risk users: > 7 drinks/week or ≥ 5 drinks on a typical drinking day or using ≥ 3 drinks several times/week. |
| Ivan et al.  2014 [69]  USA | Prevalence of alcohol consumption in older adults with anxiety | N = 223  53.4% women  ≥ 60 years with DSM-IV generalized anxiety disorder  Mean age 66.9 (± 6.6) | Cross-sectional study.  Baseline data from a randomized controlled trial (2008-12). Participants recruited from two diverse healthcare settings. | Self-reporting.  Assessed alcohol use (frequency and number of drinks consumed in an average week) during the prior month. Did not define alcohol content (g) in one drink. | Moderate drinking: ≤ 7 drinks/week  At-risk drinking: 8-14 drinks/week  Heavy drinking: > 14 drinks/week |
| Jentsch et al.  2017 [70]  Germany | Patterns of modifiable risk factors in older women | N = 4,617  100% women  ≥ 65 years | Cross-sectional study. Data from the German Health Update (2009 and 2010). Community dwelling older adults. Telephone survey. | Self-reporting.  Assessed high risk alcohol consumption with the AUDIT-C. Three questions on alcohol consumption covering drinking frequency, quantity, and how often drinking ≥ 6 units on one occasion. Score from 0-12.  Recall period not reported. | High-risk alcohol consumption:  AUDIT-C score ≥ 4 |
| Johannessen et al.  2017 [71]  Norway | Alcohol and psychotropic drug use among patients admitted to a department of old-age psychiatry | N = 206  66.5% women  ≥ 65 years  Mean age 74.8 (± 7.3) | Cross-sectional study.  Data from 12 Norwegian departments of old-age psychiatry (2013-15). Non-randomly sample.  Face-to-face interview. | Self-reporting.  Assessed alcohol consumption by the AUDIT and AUDIT-C.  Recall period not reported. | Elevated use of alcohol:  AUDIT-C score ≥ 3 in women and ≥ 4 in men |
| Kim et al.  2015 [72]  Korea | Association between alcohol consumption and depression in older adults | N = 1,819  64.8% women  ≥ 60 years  Mean age 75.6 (±7.8)  Age range 60-105 | Cross-sectional study.  Data from the Gangneung Health Study (2002-07). Random sampling. Community dwelling adults. Face-to-face interview. | Self-reporting.  Assessed alcohol consumption with AUDIT. Score from 0 to 40.  Recall period not reported. | Abstainers  No alcohol problem:  AUDIT score < 12  Problem drinkers:  AUDIT score ≥ 12 |
| Kim et al.  2020 [73]  Korea | Association between moderate alcohol intake and vivo amyloid-beta deposition in human brain | N = 414 without dementia  n = 280 cognitively normal  n = 134 mild cognitive impairment  56.5% women  Age range: 56-90  Mean age 70.9 ± 7.8 | Cross-sectional study.  Data from the Korean Brain Aging Study for Early Diagnosis and Prediction of Alzheimer’s Disease (KBASE) (2016).  Participants recruited from 2 public centers for dementia and memory clinics at two hospitals. Interviewed by trained nurses. | Self-reporting.  Systematically interviewed on current and past alcohol intake.  Assessed the amount of current drinking during the past year: standard drink(s)/day x drinking frequency/week = standard drink(s)/week.  Assessed lifetime drinking status (non-drinker, former-drinker, and drinker) and the amount of lifetime drinking: standard drink(s)/week x year = standard drink(s)/week. Age of drinking onset and age of drinking stop.  1 standard drink = 10 g pure alcohol.  1 can of beer (4.5% alcohol; 330 ml) = 1 standard drink; 1 bottle of beer (4.5% alcohol; 640 ml) = 2 standard drinks; 1 bottle of local Korean spirit (20% alcohol; 360 ml) = 6 standard drinks: 1 bottle of spirit (40% alcohol; 750 ml) = 24 standard drinks; 1 bottle of Korean traditional wine (8% alcohol; 900 ml) = 6 standard drinks; and 1 bottle of wine (12% alcohol; 900 ml) = 9 standard drinks. | Non-drinker  Former drinker: Used to drink regularly but have not drunk in the past year  Binge drinking: > 6 standard drinks per drinking day past year  Mild drinking: > 1 standard drink/week  Moderate drinking: 1-13 standard drinks/week, 10-130 g/week  Unsafe drinking (according to UK Department of Health guidelines):  ≥ 14 standard drinks/week, ≥ 140 g/week |
| Kohno et al.  2019 [74]  Japan | Association between alcohol intake and varicose veins in older adults | N = 1,060  Participants with varicose veins:  70% women  Mean age: 71.6 (±7.1)  Participants without varicose veins:  56.4% women  Mean age: 69.7 (±8.7) | Cross sectional study.  Data from the Shimane CoHRE Study (2012 and 2016).  Face-to-face interviews.  Study setting and participants not clearly described. | Self-reporting.  Assessed weekly drinking frequency and the amount (g/day) of alcohol consumed in a day.  Assessed types of alcohol: shochu, beer, sake, and others. Those who chose “others” were asked about the types of alcohol.  Calculated the amount (g/day)  according to the formula: amount (ml) x specific gravity of alcohol (0.8 g/ml) x alcohol content (beer 5%; sake 15%; shochu 35%; whiskey 43%; plum wine 20%; wine 12%).  Recall period not reported. | According to drinking frequency:  Occasional drinkers: including nondrinkers and < 1 day/week  Regular drinkers: 1–5 days/week Habitual drinkers: 6–7 days/week  According to volume:  Non-drinkers  Mild drinkers: < 20 g/day  Moderate drinkers: 20-39.9 g/day Heavy drinkers: ≥ 40 g/day |
| Lasebikan et al.  2015 [75]  Nigeria | Prevalence of alcohol consumption in older adults | N = 2,007  52.7% women  ≥ 65 years | Cross-sectional study.  The Ibadan study of Aging (2003-04). Stratified sampling.  Community dwelling older adults.  Face-to-face interview | Self-reporting.  Asked about lifetime alcohol use and past 7 days alcohol use.  1 standard unit = 15 g of alcohol | Excessive alcohol consumption:  > 1 unit/day at a sitting or > 7 units/week |
| Li et al.  2017 [76]  China and Norway | Factors associated with alcohol consumption in older adults | China:  N = 16,255  57.6% women  ≥ 65 years  Mean age 87.4 (± 11.4)  Norway:  N = 11,545  52.7% women  ≥ 65 years  Mean age 73.7 (± 6.3)  Age range 65-101 | Cross-sectional study.  Data from The Chinese Longitudinal Healthy Longevity Survey (2008-09) and The Nord-Trøndelag Health Study (HUNT3, 2006-08, Norway). Community dwelling older adults. | Self-reporting.  Assessed present alcohol consumption.  In China participants were asked if they drank alcohol at present.  In Norway participants were asked about drinking frequency last 12 months. | Consumption of alcohol:  China: Drank alcohol at present  Norway: Alcohol consumption ≥ once a month |
| Li et al.  2019 [77]  China and Norway | Factors associated with elevated alcohol consumption in older adults - comparison between China and  Norway | ≥ 65 years  China: n = 3,223  19% women  Norway: n = 6,210  43% women | Cross sectional study.  China: Chinese Longitudinal Healthy Longevity Survey data, 2008–2009. Community dwelling older adults. In-home interview.  Norway: Nord-Trøndelag Health Study data, HUNT3 2006–2008. Community dwelling older adults. The participants met at an examination station. | Self-reporting.  China: 1) Assessed alcohol consumption at present; 2) What kind of alcohol consumed (strong liquor, weak liquor, grape wine, rice wine, beer, and others); and 3) How much alcohol consumed on average per day. Calculated the number of consumed drinks of alcohol per day based on the beverage type and amount. Alcohol content by volume typically seen in China: strong liquor 53%, weak liquor 38%, grape wine 12%, rice wine 15%, and beer 4%. A Chinese unit of alcohol is called ‘Liang’ = 50 g. Recall period not reported.  Norway: Assessed drinking frequency. Recall period last 12 months. | China:  Elevated alcohol consumption:  > 1 drink/day or > 7 drinks/week  Norway:  Elevated alcohol consumption:  Drinking 4–7 days a week |
| Lima et al.  2009 [78]  Brazil | Association between alcohol consumption and falls in older adults | N = 432  59.9% women  ≥ 60 years  Mean age 69.5 (± 7.6) | Cross-sectional study.  Data from a sample of Metropolitan Säo Paulo, Brazil (2005-07). The study was a part of an international study. General population. Randomized sample. Community dwelling adults. | Self-reporting.  Assessed drinking frequency, beverage type, and consumption amounts. Estimated grams of ethanol consumed per day.  1 standard drink = 13 g of alcohol.  Recall period not reported. | Abstainers: Did not drink at all during the previous year  Light consumption: 1-2 drinks/day  Moderate consumption: 3-4 drinks/day  Heavy consumption: ≥ 5 drinks/day |
| Listabarth et al.  2021 [79]  12 European countries | Hazardous alcohol consumption among older adults | N = 13,351  57.5% women  Mean age: 72.4 (±8.4) | Cross-sectional study.  Data from the Survey of Health, Aging, and Retirement in Europe (SHARE, Wave 7: 2017 and 2018).  Computer-assisted personal interview. Community dwelling older adults. | Self-reporting.  Assessed number of alcoholic drinks consumed during the last 7 days and the frequency of excessive drinking last 3 months.  One alcoholic drink = 10 g pure alcohol. Equivalent to a can of beer (33 cl), a glass of wine (12 cl), or a shot glass of spirituous (4 cl). | Excessive drinking: ≥ 6 alcoholic drinks per occasion last 3 months  - Not at all in last 3 months  - Less than once a month  - Once or twice a month  - Once or up to 4 days a week  - 5 days a week or up to daily |
| Machado et al.  2017 [80]  Five Latin American countries | Alcohol and tobacco consumption in older couples | N = 1,451 couples (married to each other)  ≥ 65 years  Mean age 74.8 (± 6.6) | Cross-sectional study.  Data from the 10/66 Dementia Research Group (2003-07). Community dwelling older adults. | Self-reporting.  Assessed amount and frequency of maximum regular consumption of standard alcohol units in an average week. Categorized according to NIAAA guidelines for safe drinking. Did not define alcohol content (g) in one unit.  Recall period not reported. | No drinking  Any drinking: ≥ 1 unit/week  Moderate drinking: 1-7 units/week  Risk drinkers: ≥ 8 units/week |
| Marti et al.  2015 [81]  USA | Association between alcohol consumption and mental health in older adults | N = 11,191  56.4% women  ≥ 65 years | Cross-sectional study.  Data from the National Survey on Drug Use and Health (2008-12). Population based survey. Probability sampling. Community dwelling older adults. Computer assisted interview in participants residence. | Self-reporting.  Assessed ever use of alcohol, time since last drink, total number of days used alcohol, and binge drinking in the preceding 30 days. Did not define alcohol content (g) in one drink. | Lifetime abstainers  Ex-drinkers: Drank previously but not in the past 12 months.  Binge drinkers: ≥ 5 drinks on the same occasion in at least one day in the past 30 days.  Non-binge drinkers: Used alcohol more than 30 days ago, but within the past 12 months, or consumed alcohol in past 30 days, but did not binge drinking. |
| McClure et al.  2013 [82]  USA | Prevalence of risky drinking in older adults | N = 242,451  Included both women and men  ≥ 65 years | Cross-sectional study.  Data from the National Health Interview Survey (1997-2010).  Community dwelling older adults. | Self-reporting.  Assessed ever use of alcohol. Assessed alcohol consumption last 12 months. Assessed quantity consumed per day they drank alcohol and binge drinking past year.  Did not define alcohol content (g) in one drink. | Risky drinking:  Men: ≥ 10 drinks/week  Women: ≥ 7 drinks/week OR  ≥ 5 drinks at one sitting ≥ 1 time/year for both men and women |
| Moore et al.  2009 [83]  USA | Prevalence of alcohol consumption in older adults | N = 8,205  58% women  ≥ 65 years | Cross-sectional study.  Data from the National Epidemiologic Survey (2001-02). Population-based survey. Community dwelling older adults. Face-to-face interview. | Self-reporting.  Assessed lifetime and previous-12-months alcohol use. Assessed quantity and frequency of alcohol consumption. Used two definitions to define heavy drinker. Did not define alcohol content (g) in one drink. | Light drinker: ≤ 3 drinks/week  Moderate drinkers:  Men: 4-14 drinks/week  Women: 4-7 drinks/week  Definition 1: Heavy drinkers:  Men: > 14 drinks/week  Women: > 7 drinks/week  Definition 2: Heavy drinkers:  > 7 drinks/week for women and men |
| Muñoz et al.  2018 [84]  Six European countries (Germany, Italy, England, Spain, Switzerland, Israel) | Patterns of alcohol consumption in older European population | N = 3,142  50.6% women  ≥ 65 years  Mean age 73.7  Age range 65-84 years | Cross-sectional study.  Data from the MentDIS_ICF65+ study (2011). Representative sample. Community-dwelling older adults. Computer-assisted face-to-face interview. | Self-reporting.  Assessed alcohol consumption with an age sensitive diagnostic interview (Composite International Interviews [CIDI 65+]). Identified the current, 12 months and lifetime prevalence of abuse, dependence, and alcohol use disorder (AUD) according to DSM-IV. Quantified the amount of alcohol consumed on a standard day.  1 drink = 10 g of alcohol. | Lifetime drinkers: Drinking any type of alcoholic beverages ≥ 12 times during their lifetime  Abuse: Current, 12-month, and lifetime according to DSM-IV  Dependence: Current, 12-month, and lifetime according to DSM-IV  AUD: Current, 12-month, and lifetime according to DSM-IV |
| Nadkarni et al.  2011 [85]  Dominican Republic | Association between heavy drinking and psychological health of older adults’ co-residents | N = 1,391  65.1% women  ≥ 65 years | Cross-sectional study.  Data from the 10/66 dementia research group population-based research program (years not reported). Community dwelling older adults. Face-to-face to interview. | Self-reporting.  Assessed weekly alcohol consumption. Used the recommended safe weekly drinking limits for younger adults to define heavy drinking in older adults. Recall period not reported.  Did not define alcohol content (g) in one unit. | Heavy drinking:  Men: > 21 units/week  Women: > 14 units/week |
| Nogueira et al.  2013 [86]  Brazil | Prevalence of lifetime alcohol misuse in older adults | N = 1,074  71.7% women  ≥ 60 years  Mean age 71.8  Age range 60-95 | Cross-sectional study. Included representative group of participants from a state capital in South Brazil (2005-06).  Community-dwelling older adults. Face-to-face interview. | Assessed lifetime alcohol misuse with questions from the five-item Self-Reporting Questionnaire (SRQ) on lifetime alcohol abuse and misuse.  Recall period not reported. | Lifetime alcohol misuse:  SRQ score ≥ 1  Major lifetime alcohol misuse:  SRQ score ≥ 2 |
| Nuevo et al.  2015 [87]  14 European countries | Patterns of alcohol consumption in older adults | N = 10,119  61.9% women  ≥ 60 years  Mean age 70.4 (± 7.1) | Cross sectional study.  Data from the World Health Organization World Health Survey (2002-04). Randomly selected sample. Community dwelling older adults.  Face-to-face interview. | Self-reporting.  Questions about standard drink units taken during the week before the interview according to the “timeline-follow-back” method. Depending on the country, the amount of ethanol in 1 standard drink unit varied between 8 and 13 g. Interviewers showed the participants a card with pictures representing 1 standard drink unit according to the standard for each country. | Abstinence: No consumption  Occasional drinkers: At least 1 standard drink unit, but < 15 (12 for women) in a single week or < 5 (4 for women) on the same day  Frequent drinkers: ≥ 15 (≥ 12 for women) standard drink units in the entire week, but no more than 5 (4 for women) on the same day.  Heavy occasional drinkers: ≥ 5 (≥ 4 for women) standard drink units in one day, but no more than 15 (12 for women) standard drink units in the entire week  Heavy drinkers: ≥ 15 standard drink units (≥ 12 for women) during the week, and ≥ 5 (≥ 4 for women) on at least 1 day |
| Parikh et al.  2015 [88]  USA | Predictors of binge drinking in older adults | N = 4,815  52.9% women  ≥ 65 years  Mean age 71-74 (± 6-7) | Cross-sectional study.  Data from the Centers for Disease Control’s Behavioral Risk Factor Surveillance Survey (2008). Nationally representative study. Community dwelling older adults. | Self-reporting.  Assessed self-reported binge drinking. Did not define alcohol content (g) in one drink.  Recall period last month. | Binge drinking: ≥ 5 drinks for men, ≥ 4 drinks for women on at least one occasion (at the same time or within a couple of hours apart) in the past 30 days. |
| Rao et al.  2015 [89]  United Kingdom (UK) | Association between alcohol consumption, health, and socioeconomic deprivation in older adults | N = 27,991  54% women  ≥ 65 years  Mean age 74.7 (± 7.6) | Cross-sectional study.  Data from an inner-city population using a primary database from general practitioners (Lambeth DataNet) (2013). Sample not representative of the UK population. Community dwelling older adults. | Self-reporting.  Assessed volume of alcohol consumption (units per week). Classified unsafe drinkers according to recommended safe limits. Did not define alcohol content (g) in one unit.  Recall period not reported. | Unsafe drinkers:  Men: > 21 units/week  Women: > 14 units/week |
| Roson et al.  2010 [90]  Spain | Prevalence of unhealthy alcohol use in hospitalized patients | N = 1,039  47% women  ≥ 18 years  Mean age 72.1 | Cross-sectional study.  Data assessed from hospitalized population in General Internal Medicine Wards in 21 hospitals (2008). | Self-reporting.  Assessed alcohol consumption and risky alcohol consumption with AUDIT-C and AUDIT, and the Systematic Inventory of Alcohol consumption questionnaire (SIAC). Did not define alcohol content (g) in one drink.  Recall period not reported. | Abstainers: Did not drink alcoholic beverages (score 0 on AUDIT-C or with negative response to the first question)  Current drinkers: > 1 drink/week  Low risk drinkers:  Positive response to the first question in AUDIT-C and SIAC  Men: ≤ 280 g/week or ≤ 28 standard drinks/week  Women: ≤ 140 g/week or ≤ 14 standard drinks/week  AUDIT-C score < 4 in men and < 3 in women  Risky alcohol consumption:  Men: > 280 g/week or > 28 standard drinks/week  Women: > 140 g/week or > 14 standard drinks/week  Increased risk of hazardous drinking/alcohol abuse or dependence: AUDIT-C score ≥ 4 in men and ≥ 3 in women  Hazardous and harmful drinking:  AUDIT-score ≥ 8 in men and ≥ 6 in women |
| Ryan et al.  2013 [91]  USA | Prevalence of alcohol consumption in older adults with chronic medical conditions | N = 7,422  59.5% women  ≥ 65 years | Cross-sectional study.  Data from the Medicare Current Beneficiary Survey linked with Medicare claims (2005). Community-dwelling older adults with ≥ 1 of 7 chronic conditions. In-person interviews. | Self-reporting.  Assessed drinking frequency and how many drinks they consumed the day they drank alcohol. Assessed how many days they had ≥ 4 drinks in a single day. Did not define alcohol content (g) in one drink. Categorized drinking pattern according to NIAAA recommended guidelines.  Recall period 12 months. | Exceeding monthly limits: > 30 drinks per typical month  Heavy episodic drinking: ≥ 4 drinks in any single day  Within guidelines drinkers: Not exceeding the monthly limit (≤ 30 drinks per typical month) or the single day limit (< 4 drinks in any single day).  At-risk drinkers: exceeded guidelines: Exceeded the monthly limit, but not the single-day limit, and heavy episodic drinkers who exceeded the single-day drinking limit with or without exceeding the monthly limit. |
| Sacco et al.  2009 [92]  USA | Examined alcohol use in older adults | N = 4,646  47.9% women  ≥ 60 years  Mean age 70.1 | Cross-sectional study.  Data from the National Epidemiologic Survey on Alcohol and Related Conditions (Wave 1, 2001-2002). Nationally representative sample. Community dwelling older adults.  In-person interviews. | Self-reporting.  Assessed alcohol consumption and alcohol disorders using the Alcohol Use Disorder and Associated Disabilities Interview Schedule – DSM-IV version. Assessed quantity and frequency of alcohol consumption. Did not define alcohol content (g) in one drink. Drinking pattern categorized according to NIAAA guidelines.  Recall period 12 months. | At risk consumption: > 7 drinks/week  Heavy episodic use: ≥ 5 drinks on one occasion in the past year  Past-year alcohol abuse or dependence: Defined according to DSM-IV  Low-risk drinker class: More likely to exceed consumption guidelines  High-risk drinker class: High probability of both to exceed consumption guidelines and to have DSM-IV criteria. |
| Sanford et al.  2020 [93]  USA | Alcohol use among patients with cancer and survivors | N = 34,080  59.5% women  Median age: 67  Age range: 55-76 | Cross sectional study.  Data from the  National Health Interview Survey (2000 to 2017).  Household survey.  Noninstitutionalized adults. | Self-reporting.  Assessed current alcohol use.  Asked the participants about the average number of drinks consumed on days they drank and how many days during the past year they had consumed ≥ 5 drinks.  Did not define alcohol content (g) in one drink. | Lifetime abstainers: < 12 drinks in life  Former drinkers: No drinks in the past year  Current drinkers: ≥ 1 drinks in the past year  Exceeding moderate drinking limits: > 1 drink/day for women and > 2 drinks/day for men (CDC guidelines)  Binge drinking:  ≥ 5 drinks during at least one day over the past year in women and men |
| Satre et al.  2011 [94]  USA | Examined patterns of alcohol and drug use among depressed older adults | N = 154  62.4% women  ≥ 60 years (with mild or greater symptoms of depression)  Mean age 67.6 | Cross-sectional study.  Data from patients seeking out-patient services at an adult Psychiatric clinic (2005-2009).  Telephone interview.  . | Self-reporting.  Assessed ever use of alcohol, usual quantity (in standard drinks), frequency in the prior 30 days, and heavy episodic drinking in the prior year based on the graduated frequency alcohol consumption measurement method. Did not define alcohol content (g) in one drink. Assessed alcohol problem with the Short Michigan Alcohol Screening Test (SMAST), self-administered scale. Score 0-13. | Usual quantity of drinks per month:  standard drinks/month  Drinking frequency per month: days/month  Heavy episodic drinking:  ≥ 5 (5-7 or ≥ 8) on ≥ 1 occasions in the prior year  Possible lifetime alcohol problems: SMAST score ≥ 3 |
| Suo et al.  2019 [95]  China | Association between alcohol variants of genes encoding alcohol metabolism enzymes, and risk of esophageal cancer | Cases: n = 1,190 cases with esophageal cancer  34% women  Mean age: 66.9  Age range: 42-85  Controls: n = 1,883  32% women  Mean age: 66.4  Age range: 40-85 | Case-control study with cross-sectional design.  Population based study.  Included patients diagnosed with esophageal cancer between 2010-2013.  > 90% of the patients recruited from four hospitals. Each patient completed an electronic questionnaire by  trained interviewers. | Self-reporting.  Asked at what age participants started drinking, at what age they stopped drinking, the amount they typically drank, types of alcohol, how often they drank, and the number of years for which they had consumed alcohol. Amount of alcohol consumed were measured in cups for Chinese and imported spirits and in bottles for beer, fruit wine, yellow rice wine, and grape wine. The amount of alcohol converted from cups/bottles to grams. Did not define alcohol content (g) in one drink. | Ex-drinkers: stopped drinking alcohol for at least 2 years before the interview date  Cumulative drinking amount (g/day-years):  - Never  - < 1000  - 1000-2250  - 2250-4000  - ≥ 4000 |
| Tevik et al.  2017 [96]  Norway | Prevalence of alcohol consumption and use of drugs with addiction potential in older adults | N = 11,545  52.7% women  ≥ 65 years  Mean age 73.7 (± 6.3) | Cross-sectional study.  Data from The Nord-Trøndelag Health Study (HUNT3, 2006-08). Population based study.  Community-dwelling older adults. | Self-reporting.  Assessed drinking frequency last 12 months. | Non-regular drinker: < 1 day/week  Regular drinkers: ≥ 1 day/week  Regular drinkers categorized according to drinking frequency:  1 day/week  2-3 days/week  ≥ 4 days/week |
| Towers et al.  2018 [97]  New Zealand | Association between alcohol consumption and health in older adults | N = 2,976  52% women  ≥ 52 years  Mean age 65  Age range 52-86 | Cross-sectional study.  Data from the New Zealand Longitudinal Study of Ageing (2012). Community dwelling older adults. Postal questionnaire. | Self-reporting.  Assessed alcohol consumption with the first two items of the AUDIT-C (frequency and quantity of consumption). Calculated average daily drinking amount. Did not define alcohol content (g) in one drink.  Recall period not reported. | Lifetime abstainers  Current non-drinkers  Less than 1 drink/daily  Moderate drinkers: 1-2 drinks/day  Heavy drinkers: ≥ 3 drinks/day |
| Towers et al.  2019 [98]  New Zealand | Prevalence of hazardous drinking: comparison of the AUDIT-C and the CARET | N = 3,673  49% women  Age range: 55 to 89  Mean age: 65.5 (±7) | Cross-sectional study.  Data from the New Zealand Health, Work and Retirement Study (2016). Postal survey. Equal probability random sampling. General population. Community dwelling older adults. | Self-reporting.  Assessed alcohol consumption with the screening tools AUDIT-C and CARET.  AUDIT-C consists of three questions assessing frequency and quantity of alcohol use, and frequency of binge drinking (i.e., 6+ standards  drinks per occasion) in the past 12 months. Total score 0-12.  CARET: 27 items. Assessing frequency, quantity, and binge drinking. Assessing whether drinking occur in the presence of i.e., comorbidities, alcohol-interactive medication, and alcohol risk behaviors. | Differentiated between lifetime abstainers and current abstainers.  AUDIT-C  - Non-hazardous drinking  - Hazardous drinking (score of 4  for men and 3 for women)    CARET:  - Non-hazardous drinking  - Hazardous drinking  (cut-off value not specified) |
| Vafeas et al.  2017 [99]  Australia | Examined patterns of alcohol consumption in older adults | N = 1,550  Included both women and men  ≥ 65 years | Cross-sectional study.  A repeated cross-sectional survey design over a 4-year period.  Data from the South West Senior’s Expos (2011-14). Community-dwelling older adults. | Self-reporting.  Assessed risk of harmful or hazardous alcohol consumption with AUDIT (10 questions).  Recall period not reported. | Low risk: AUDIT score 0-7  Risky alcohol use: AUDIT score 8-12  High risk alcohol use: AUDIT score ≥ 13  High consumption risk:  Score ≥ 6 for question 1-3 may indicate risk of alcohol-related harm  High dependence risk:  Score ≥ 4 for question 4-6, possibility of alcohol dependence  High alcohol-related problems risk: Any score > 0 for question 7-10 |
| Villar Luis et al.  2018 [100]  Brazil | Examined patterns of alcohol consumption in older adults | N = 25  56% women  ≥ 60 years  Mean age 69.8  Age range 60-83 | Cross-sectional study.  Data from a community service, the Family Health Center (2016). Included participants with AUDIT score ≥ 7 and MAST-G score ≥ 5. Community-dwelling older adults. Interviewed in their home. | Self-reporting.  Assessed alcohol consumption with AUDIT (10 questions) and MAST-G (24 questions).  Recall period not reported. | Low risk use: AUDIT score 0-7  Risk use: AUDIT score 8-14  Alcohol related problems: MAST-G score ≥ 5 |
| Waern et al.  2014 [101]  Sweden | Prevalence of at-risk drinking in two birth cohorts of older adults | N = 1,056  55.7% women  ≥ 75 years  75-years-olds born in 1901-02 (n = 303) and in 1930 (n = 753) | Cross-sectional study.  Representative sample of an older birth cohort in Gothenburg. 75-year-olds examined in 1976–77 and in 2005–06. | Self-reporting.  Psychiatric interview.  Assessed drinking frequency and total weekly alcohol intake and converted the consumption into grams of alcohol. The following conversion factors were used: beer: 0.33, wine: 1 and spirits: 3. | Abstainers  Drinking frequency by beverage type: (beer, wine, spirits)  - None in latest month  - ≤ 2 times/week  - ≥ 3 times/week  Mean alcohol consumption:  g alcohol/week  Low-moderate drinking levels:  0-100 g alcohol/week  At-risk drinking: ≥ 100 g alcohol/week in men and women  Alternative cut-off for at-risk drinking in women: ≥ 60 g alcohol/week |
| Wang et al.  2017 [102]  China | Gender difference in mental health, smoking, drinking, and chronic diseases in older adults. | N = 4,115  53.4% women  ≥ 60 years  Mean age 65.8 (± 4.9)  Age range 60-79 | Cross-sectional study.  Data from the Jilin Provincial Chronic Disease survey (2012). Community-dwelling older adults.  Face-to-face interview. | Self-reporting.  Assessed current alcohol consumption and drinking frequency last year before the survey. | Current drinking: Alcohol consumption last year  Frequent drinking: Drinking 5-7 days/week |
| Weyerer et al.  2009 [103]  Germany | Prevalence of at-risk drinking in older adults | N = 3,224  65.7% women  ≥ 75 years  Mean age 80.2 (± 3.6)  Age range 75-99 | Cross-sectional study.  Data from individuals attending general practitioners in Germany. Interviewed in their homes (2003 and 2004). Community dwelling older adults. | Self-reporting.  Frequency of alcohol consumption and quantity of beer, wine, and liquor was determined. Did not describe the method used to convert the quantity of drinks to grams of alcohol. Defined risk drinking according to the guidelines of the British Medical Associations.  Recall period not reported. | Abstainers  Moderate drinkers:  Men: ≤ 30 g alcohol/day  Women: ≤ 20 g alcohol/day  At-risk drinkers:  Men: > 30 g alcohol/day  Women: > 20 g alcohol/day |
| Wilson et al.  2014 [104]  USA | Prevalence of harmful and hazardous alcohol consumption in older adults | N = 36,136,889  57.5% women  ≥ 65 years | Cross-sectional study.  Data from the National Health and Nutrition Examination Survey (2005-06 and 2007-08). Community dwelling older adults. | Self-reporting.  Classified different patterns of alcohol consumption according to the Alcohol-Related Problems Survey (ARPS) and NIAAA recommendation for alcohol consumption. Assessed the quantity and frequency of alcohol consumption (past 12 months) in relation to 63 factors, i.e., medical problems, medication use, binge drinking. ARPS Risk Classification Algorithm were used for screening for harmful and hazardous consumption. Did not define alcohol content (g) in one drink. | Light/moderate alcohol consumption:  ≤ 7 drinks/week, and  ≤ 3 drinks/day  Frequency of alcohol consumption:  Never, ≤ 1 time a month, 2-4 times a month, 2-3 times a week, 4-5 times a week, daily/almost daily  Quantity consumed:  ≤ 1, 2, 3, 4, ≥ 5 drinks/day  Alcohol in excess of NIAAA limits:  > 7 drinks/week  > 3 drinks/day  Binge drinking: ≥ 5 drinks in at least 1 day in the past 12 months.  Harmful consumption: According to ARPS classification.  Hazardous consumption: According to ARPS classification. |
| Zaitsu et al.  2020 [105]  Japan | Association between light to moderate lifetime alcohol consumption  and cancer | N = 126,464  34.7% women  ≥ 20 years  Mean age: 69 (± 10)  Cancer cases:  n = 63,232  Hospital controls:  n = 63,232 | Case-control study with cross-sectional design.  Data from a nationwide, hospital-based data set (ICOD-R: 2005-2016). A multicenter study. Included patients who had been admitted to the hospital between 2005-2016. Assessed data at admission or during hospital stay. | Self-reporting.  Assessed average daily amount of standardized alcohol units and duration of drinking (years). Multiplied the daily amount of standardized alcohol use (drinks per day) and the duration of drinking (years).  One standard drink = 23 g ethanol. Equivalent to one 180-ml cup (6 ounces) of Japanese sake, one 500-ml bottle (17 ounces) of beer, one 180-ml glass (6 ounces) of wine, or one 60-ml cup (2 ounces) of whiskey. | Drinking history:  Never, Former, and Current drinker  Duration of drinking:  Never  0-19 year  20-39 year  ≥ 40 years  Categorized 6 categories by their drink-year levels:  0 (lifetime abstainer = never consumed alcohol)  > 0-20 drink-years  > 20-40 drink-years  > 40-60 drink-years  > 60-90 drink-years  > 90 drink-years  Alternative 7 drinking categories:  0 drinks per day (lifetime abstainer)  ≤ 2 drinks/day and < 20 years  ≤ 2 drinks/day and 20-39 years  ≤ 2 drinks/day and ≥ 40 years,  > 2 drinks/day and < 20 years,  > 2 drinks/day and 20-39 years  > 2 drinks/day and ≥ 40 years |

AUDIT = Alcohol Use Disorders Identification Test; CAGE = Cut down, Annoyed, Guilty, Eye opener; CARET = Comorbidity Alcohol Risk Evaluation Tool; DSM-IV = Diagnostic and Statistical Manual of Mental Disorders, 4th Edition; CDC = Center for Disease Control and Prevention; ICD-10 = International Classification of Diseases 10th Revision; g = gram; MAST-G = Michigan Alcoholism Screening Test – Geriatric Version; NIAAA = National Institute on Alcohol Abuse and Alcoholism

**References**

1. Agahi N, Kelfve S, Lennartsson C, Kareholt I. Alcohol consumption in very old age and its association with survival: A matter of health and physical function. Drug and alcohol dependence. 2016;159:240-5. Epub 2016/01/18. doi: 10.1016/j.drugalcdep.2015.12.022. PubMed PMID: 26775285.

2. Agahi N, Dahlberg L, Lennartsson C. Social integration and alcohol consumption among older people: A four-year follow-up of a Swedish national sample. Drug & Alcohol Dependence. 2019;196:40-5. doi: 10.1016/j.drugalcdep.2018.12.011. PubMed PMID: 134662997.

3. Aguila E, Guerrero EG, Vega WA. Sociodemographic characteristics associated with alcohol use among low-income Mexican older adults. Substance Abuse Treatment, Prevention & Policy. 2016;11:1-9. doi: 10.1186/s13011-016-0061-6. PubMed PMID: 115255260.

4. AlGhatrif M, Markides KS, Kuo Y-f, Ray LA, Moore AA. The effect of prevalent cardiovascular conditions on the association between alcohol consumption and mortality among older Mexican American men. Ethnicity & disease. 2013;23(2):168-74. PubMed PMID: 107998758.

5. Almeida OP, Hankey GJ, Yeap BB, Golledge J, Flicker L. Alcohol consumption and cognitive impairment in older men: A mendelian randomization study. Neurology. 2014;82(12):1038-44. doi: 10.1212/WNL.0000000000000255. PubMed PMID: 107896468.

6. Almeida OP, McCaul K, Hankey GJ, Yeap BB, Golledge J, Flicker L. Excessive alcohol consumption increases mortality in later life: a genetic analysis of the health in men cohort study. Addiction biology. 2017;22(2):570-8. doi: 10.1111/adb.12340. PubMed PMID: 121408259.

7. Bazal P, Gea A, Martínez-González MA, Salas-Salvadó J, Asensio EM, Muñoz-Bravo C, et al. Mediterranean alcohol-drinking pattern, low to moderate alcohol intake and risk of atrial fibrillation in the PREDIMED study. Nutrition, metabolism, and cardiovascular diseases : NMCD. 2019;29(7):676-83. Epub 2019/05/13. doi: 10.1016/j.numecd.2019.03.007. PubMed PMID: 31078364.

8. Bell S, Britton A. Reliability of a retrospective decade-based life-course alcohol consumption questionnaire administered in later life. Addiction (Abingdon, England). 2015;110(10):1563-73. Epub 2015/06/09. doi: 10.1111/add.13012. PubMed PMID: 26052751; PubMed Central PMCID: PMCPMC4587356.

9. Britton A, Fat LN, Neligan A. The association between alcohol consumption and sleep disorders among older people in the general population. Scientific reports. 2020;10(1):5275. Epub 2020/03/27. doi: 10.1038/s41598-020-62227-0. PubMed PMID: 32210292; PubMed Central PMCID: PMCPMC7093458.

10. Buja A, Scafato E, Baggio B, Sergi G, Maggi S, Rausa G, et al. Renal impairment and moderate alcohol consumption in the elderly. Results from the Italian Longitudinal Study on Aging (ILSA). Public health nutrition. 2011;14(11):1907-18. Epub 2011/07/07. doi: 10.1017/s1368980011000863. PubMed PMID: 21729477.

11. Buja A, Scafato E, Sergi G, Maggi S, Suhad MA, Rausa G, et al. Alcohol consumption and metabolic syndrome in the elderly: results from the Italian longitudinal study on aging. European journal of clinical nutrition. 2010;64(3):297-307. Epub 2009/11/26. doi: 10.1038/ejcn.2009.136. PubMed PMID: 19935817.

12. Chavez LJ, Liu C-F, Tefft N, Hebert PL, Clark BJ, Rubinsky AD, et al. Unhealthy alcohol use in older adults: Association with readmissions and emergency department use in the 30 days after hospital discharge. Drug & Alcohol Dependence. 2016;158:94-101. doi: 10.1016/j.drugalcdep.2015.11.008. PubMed PMID: 111928578.

13. Cohen-Mansfield J. Trends in Health Behaviors in the Old-Old Population: Results from a National Survey. Behavioral Medicine. 2012;38(1):6-11. doi: 10.1080/08964289.2011.644642. PubMed PMID: 104523552.

14. Dhana K, Evans DA, Rajan KB, Bennett DA, Morris MC. Healthy lifestyle and the risk of Alzheimer dementia: Findings from 2 longitudinal studies. Neurology. 2020;95(4):e374-e83. Epub 2020/06/20. doi: 10.1212/wnl.0000000000009816. PubMed PMID: 32554763; PubMed Central PMCID: PMCPMC7455318.

15. Gargiulo G, Testa G, Cacciatore F, Mazzella F, Galizia G, Della-Morte D, et al. Moderate alcohol consumption predicts long-term mortality in elderly subjects with chronic heart failure. The journal of nutrition, health & aging. 2013;17(5):480-5. Epub 2013/05/03. doi: 10.1007/s12603-012-0430-4. PubMed PMID: 23636551.

16. Goulden R. Moderate Alcohol Consumption Is Not Associated with Reduced All-cause Mortality. The American journal of medicine. 2016;129(2):180-6.e4. Epub 2015/11/03. doi: 10.1016/j.amjmed.2015.10.013. PubMed PMID: 26524703.

17. Halme JT, Seppa K, Alho H, Poikolainen K, Pirkola S, Aalto M. Alcohol consumption and all-cause mortality among elderly in Finland. Drug and alcohol dependence. 2010;106(2-3):212-8. Epub 2009/09/29. doi: 10.1016/j.drugalcdep.2009.08.017. PubMed PMID: 19782479.

18. Hassing LB. Light Alcohol Consumption Does Not Protect Cognitive Function: A Longitudinal Prospective Study. Front Aging Neurosci. 2018;10:81. Epub 2018/04/11. doi: 10.3389/fnagi.2018.00081. PubMed PMID: 29632484; PubMed Central PMCID: PMCPMC5879951.

19. Heffernan M, Mather KA, Jing X, Assareh AA, Kochan NA, Reppermund S, et al. Alcohol Consumption and Incident Dementia: Evidence from the Sydney Memory and Ageing Study. Journal of Alzheimer's Disease. 2016;52(2):529-38. doi: 10.3233/JAD-150537. PubMed PMID: 115360609.

20. Hoang TD, Byers AL, Barnes DE, Yaffe K. Alcohol consumption patterns and cognitive impairment in older women. American Journal of Geriatric Psychiatry. 2014;22(12):1663-7. doi: 10.1016/j.jagp.2014.04.006. PubMed PMID: 109767289.

21. Holton A, Boland F, Gallagher P, Fahey T, Kenny RA, Cousins G. Longitudinal prevalence of potentially serious alcohol-medication interactions in community-dwelling older adults: a prospective cohort study. European Journal of Clinical Pharmacology. 2019;75(4):569-75. doi: 10.1007/s00228-018-02608-7. PubMed PMID: 135501939.

22. Iparraguirre J. Socioeconomic determinants of risk of harmful alcohol drinking among people aged 50 or over in England. BMJ open. 2015;5(7):e007684. doi: https://dx.doi.org/10.1136/bmjopen-2015-007684. PubMed PMID: 26204909.

23. Jeong HG, Kim TH, Lee JJ, Lee SB, Park JH, Huh Y, et al. Impact of alcohol use on mortality in the elderly: results from the Korean Longitudinal Study on Health and Aging. Drug and alcohol dependence. 2012;121(1-2):133-9. Epub 2011/09/13. doi: 10.1016/j.drugalcdep.2011.08.017. PubMed PMID: 21908108.

24. Liu R, Chen L, Zhang F, Zhu R, Lin X, Meng X, et al. Trends in Alcohol Intake and the Association between Socio-Demographic Factors and Volume of Alcohol Intake amongst Adult Male Drinkers in China. International journal of environmental research and public health. 2019;16(4). Epub 2019/02/20. doi: 10.3390/ijerph16040573. PubMed PMID: 30781481; PubMed Central PMCID: PMCPMC6406336.

25. McCaul KA, Almeida OP, Hankey GJ, Jamrozik K, Byles JE, Flicker L. Alcohol use and mortality in older men and women. Addiction (Abingdon, England). 2010;105(8):1391-400. Epub 2010/06/10. doi: 10.1111/j.1360-0443.2010.02972.x. PubMed PMID: 20528808.

26. McEvoy LK, Kritz-Silverstein D, Barrett-Connor E, Bergstrom J, Laughlin GA. Changes in alcohol intake and their relationship with health status over a 24-year follow-up period in community-dwelling older adults. Journal of the American Geriatrics Society. 2013;61(8):1303-8. Epub 2013/07/20. doi: 10.1111/jgs.12366. PubMed PMID: 23865905; PubMed Central PMCID: PMCPMC3819601.

27. Merrick ES, Hodgkin D, Garnick DW, Horgan CM, Panas L, Ryan M, et al. Older adults' inpatient and emergency department utilization for ambulatory-care-sensitive conditions: relationship with alcohol consumption. Journal of aging and health. 2011;23(1):86-111. Epub 2010/10/12. doi: 10.1177/0898264310385114

10.1177/0898264310383156. PubMed PMID: 20935248; PubMed Central PMCID: PMCPMC3021178.

28. Ormstad H, Rosness TA, Bergem AL, Bjertness E, Strand BH. Alcohol consumption in the elderly and risk of dementia related death - a Norwegian prospective study with a 17-year follow-up. The International journal of neuroscience. 2016;126(2):135-44. Epub 2014/12/17. doi: 10.3109/00207454.2014.997876. PubMed PMID: 25495993.

29. Ortola R, Garcia-Esquinas E, Galan I, Guallar-Castillon P, Lopez-Garcia E, Banegas JR, et al. Patterns of alcohol consumption and risk of falls in older adults: a prospective cohort study. Osteoporosis international : a journal established as result of cooperation between the European Foundation for Osteoporosis and the National Osteoporosis Foundation of the USA. 2017;28(11):3143-52. Epub 2017/07/21. doi: 10.1007/s00198-017-4157-2. PubMed PMID: 28725986.

30. Ortolá R, García-Esquinas E, Soler-Vila H, Ordovas JM, López-García E, Rodríguez-Artalejo F. Changes in health status predict changes in alcohol consumption in older adults: the Seniors-ENRICA cohort. Journal of epidemiology and community health. 2019;73(2):123-9. Epub 2018/11/01. doi: 10.1136/jech-2018-211104. PubMed PMID: 30377248.

31. Richard EL, Kritz-Silverstein D, Laughlin GA, Fungb TT, Barrett-Connor E, McEvoy LK, et al. Alcohol Intake and Cognitively Healthy Longevity in Community-Dwelling Adults: The Rancho Bernardo Study. Journal of Alzheimer's Disease. 2017;59(3):803-14. doi: 10.3233/JAD-161153. PubMed PMID: 124462585.

32. Scott RG, Wiener CH, Paulson D. The Benefit of Moderate Alcohol Use on Mood and Functional Ability in Later Life: Due to Beers or Frequent Cheers? The Gerontologist. 2020;60(1):80-8. Epub 2018/10/26. doi: 10.1093/geront/gny129. PubMed PMID: 30358833.

33. Shaw BA, Agahi N, Krause N, Shaw BA, Agahi N, Krause N. Are changes in financial strain associated with changes in alcohol use and smoking among older adults? Journal of Studies on Alcohol & Drugs. 2011;72(6):917-25. PubMed PMID: 108200408.

34. Shiotsuki H, Saijo Y, Ogushi Y, Kobayashi S. Relationships between Alcohol Intake and Ischemic Stroke Severity in Sex Stratified Analysis for Japanese Acute Stroke Patients. Journal of stroke and cerebrovascular diseases : the official journal of National Stroke Association. 2019;28(6):1604-17. Epub 2019/03/25. doi: 10.1016/j.jstrokecerebrovasdis.2019.02.034. PubMed PMID: 30904471.

35. Siddiquee AT, Kadota A, Fujiyoshi A, Miyagawa N, Saito Y, Suzuki H, et al. Alcohol consumption and cognitive function in elderly Japanese men. Alcohol (Fayetteville, NY). 2020;85:145-52. Epub 2020/01/11. doi: 10.1016/j.alcohol.2020.01.001. PubMed PMID: 31923561.

36. Soler-Vila H, Ortolá R, García-Esquinas E, León-Muñoz LM, Rodríguez-Artalejo F. Changes in Alcohol Consumption and Associated Variables among Older Adults in Spain: A population-based cohort study. Scientific reports. 2019;9(1):10401. Epub 2019/07/20. doi: 10.1038/s41598-019-46591-0. PubMed PMID: 31320663; PubMed Central PMCID: PMCPMC6639301.

37. Tait RJ, French DJ, Burns RA, Byles JE, Anstey KJ. Alcohol, hospital admissions, and falls in older adults: a longitudinal evaluation. International psychogeriatrics. 2013;25(6):901-12. Epub 2013/02/26. doi: 10.1017/s1041610213000173. PubMed PMID: 23432881.

38. Tateishi R, Uchino K, Fujiwara N, Takehara T, Okanoue T, Seike M, et al. A nationwide survey on non-B, non-C hepatocellular carcinoma in Japan: 2011-2015 update. Journal of gastroenterology. 2019;54(4):367-76. Epub 2018/12/01. doi: 10.1007/s00535-018-1532-5. PubMed PMID: 30498904; PubMed Central PMCID: PMCPMC6437291.

39. Tevik K, Selbæk G, Engedal K, Seim A, Krokstad S, Helvik AS. Mortality in older adults with frequent alcohol consumption and use of drugs with addiction potential - The Nord Trøndelag Health Study 2006-2008 (HUNT3), Norway, a population-based study. PloS one. 2019;14(4):e0214813. Epub 2019/04/17. doi: 10.1371/journal.pone.0214813. PubMed PMID: 30990815; PubMed Central PMCID: PMCPMC6467384.

40. van Oort S, Beulens JW, van der Heijden A, Elders PJM, Stehouwer CDA, van de Luitgaarden IAT, et al. Moderate and heavy alcohol consumption are prospectively associated with decreased left ventricular ejection fraction: The Hoorn Study. Nutrition, metabolism, and cardiovascular diseases : NMCD. 2020;30(1):132-40. Epub 2019/11/02. doi: 10.1016/j.numecd.2019.09.021. PubMed PMID: 31672450.

41. Villalonga-Olives E, Almansa J, Shaya F, Kawachi I. Perceived social capital and binge drinking in older adults: The Health and Retirement Study, US data from 2006-2014. Drug and alcohol dependence. 2020;214:108099. Epub 2020/08/01. doi: 10.1016/j.drugalcdep.2020.108099. PubMed PMID: 32736315.

42. Weyerer S, Schaufele M, Wiese B, Maier W, Tebarth F, van den Bussche H, et al. Current alcohol consumption and its relationship to incident dementia: results from a 3-year follow-up study among primary care attenders aged 75 years and older. Age and ageing. 2011;40(4):456-63. Epub 2011/03/04. doi: 10.1093/ageing/afr007. PubMed PMID: 21367764.

43. Aalto M, Alho H, Halme JT, Seppä K. The alcohol use disorders identification test (AUDIT) and its derivatives in screening for heavy drinking among the elderly. International journal of geriatric psychiatry. 2011;26(9):881-5. doi: 10.1002/gps.2498. PubMed PMID: 104672180.

44. Ahlner F, Sigstrom R, Sterner TR, Fassberg MM, Kern S, Ostling S, et al. Increased alcohol consumption among Swedish 70-year-olds 1976 to 2016: Analysis of data from The Gothenburg H70 Birth Cohort Studies, Sweden. Alcoholism: Clinical and Experimental Research. 2018;42(12):2403-12. doi: http://dx.doi.org/10.1111/acer.13893. PubMed PMID: 2018-63313-013.

45. Barnes AJ, Moore AA, Xu H, Ang A, Tallen L, Mirkin M, et al. Prevalence and correlates of at-risk drinking among older adults: the project SHARE study. Journal of general internal medicine. 2010;25(8):840-6. Epub 2010/04/17. doi: 10.1007/s11606-010-1341-x. PubMed PMID: 20396975; PubMed Central PMCID: PMCPMC2896609.

46. Bryant AN, Kim G. The relation between acculturation and alcohol consumption patterns among older Asian and Hispanic immigrants. Aging & mental health. 2013;17(2):147-56. Epub 2012/10/27. doi: 10.1080/13607863.2012.727382. PubMed PMID: 23098103.

47. Bryant AN, Kim G. The relation between frequency of binge drinking and psychological distress among older adult drinkers. Journal of aging and health. 2013;25(7):1243-57. Epub 2013/08/31. doi: 10.1177/0898264313499933. PubMed PMID: 23988812.

48. Bryant AN, Kim G. Regional and racial/ethnic variations in alcohol consumption among older adults. Aging & mental health. 2019;23(11):1503-9. Epub 2019/01/03. doi: 10.1080/13607863.2018.1506746. PubMed PMID: 30600687; PubMed Central PMCID: PMCPMC6606409.

49. Chan KK, Chiu KC, Chu LW. Association between alcohol consumption and cognitive impairment in Southern Chinese older adults. International journal of geriatric psychiatry. 2010;25(12):1272-9. Epub 2010/11/19. doi: 10.1002/gps.2470. PubMed PMID: 21086537.

50. Choi NG, Dinitto DM, Choi NG, Dinitto DM. Heavy/binge drinking and depressive symptoms in older adults: gender differences. International journal of geriatric psychiatry. 2011;26(8):860-8. doi: 10.1002/gps.2616. PubMed PMID: 104657950.

51. Cousins G, Galvin R, Flood M, Kennedy MC, Motterlini N, Henman MC, et al. Potential for alcohol and drug interactions in older adults: evidence from the Irish longitudinal study on ageing. BMC geriatrics. 2014;14:57. Epub 2014/04/29. doi: 10.1186/1471-2318-14-57. PubMed PMID: 24766969; PubMed Central PMCID: PMCPMC4008399.

52. D'Ovidio F, Rooney JPK, Visser AE, Manera U, Beghi E, Logroscino G, et al. Association between alcohol exposure and the risk of amyotrophic lateral sclerosis in the Euro-MOTOR study. J Neurol Neurosurg Psychiatry. 2019;90(1):11-9. Epub 2018/08/05. doi: 10.1136/jnnp-2018-318559. PubMed PMID: 30076269.

53. Davis BJK, Vidal J-S, Garcia M, Aspelund T, van Buchem MA, Jonsdottir MK, et al. The alcohol paradox: light-to-moderate alcohol consumption, cognitive function, and brain volume. Journals of Gerontology Series A: Biological Sciences & Medical Sciences. 2014;69(12):1528-35. doi: 10.1093/gerona/glu092. PubMed PMID: 103853954.

54. Forlani M, Morri M, Belvederi Murri M, Bernabei V, Moretti F, Attili T, et al. Anxiety symptoms in 74+ community-dwelling elderly: associations with physical morbidity, depression and alcohol consumption. PloS one. 2014;9(2):e89859. Epub 2014/03/04. doi: 10.1371/journal.pone.0089859. PubMed PMID: 24587079; PubMed Central PMCID: PMCPMC3935948.

55. Foster J, Patel S. Prevalence of simultaneous use of alcohol and prescription medication in older adults: findings from a cross-sectional survey (Health Survey for England 2013). BMJ open. 2019;9(6):e023730. Epub 2019/07/01. doi: 10.1136/bmjopen-2018-023730. PubMed PMID: 31256017; PubMed Central PMCID: PMCPMC6609060.

56. Fuentes S, Bilal U, Galan I, Villalbi JR, Espelt A, Bosque-Prous M, et al. Binge drinking and well-being in European older adults: do gender and region matter? European journal of public health. 2017;27(4):692-9. Epub 2017/04/22. doi: 10.1093/eurpub/ckw246. PubMed PMID: 28431128; PubMed Central PMCID: PMCPMC5881769.

57. Gibson RC, Waldron NK, Abel WD, Eldemire-Shearer D, James K, Mitchell-Fearon K. Alcohol use, depression, and life satisfaction among older persons in Jamaica. International psychogeriatrics. 2017;29(4):663-71. Epub 2016/12/13. doi: 10.1017/s1041610216002209. PubMed PMID: 27938435.

58. González-Rubio E, San Mauro I, López-Ruíz C, Díaz-Prieto L, Marcos A, Nova E, et al. Relationship of moderate alcohol intake and type of beverage with health behaviors and quality of life in elderly subjects. Quality of Life Research. 2016;25(8):1931-42. doi: 10.1007/s11136-016-1229-2. PubMed PMID: 116816460.

59. Guidolin BL, Silva Filho IG, Nogueira EL, Ribeiro Junior FP, Cataldo Neto A. Patterns of alcohol use in an elderly sample enrolled in the Family Health Strategy program in the city of Porto Alegre, Brazil. Ciencia & saude coletiva. 2016;21(1):27-35. Epub 2016/01/28. doi: 10.1590/1413-81232015211.10032015. PubMed PMID: 26816160.

60. Hajek A, Bock JO, Weyerer S, Konig HH. Correlates of alcohol consumption among Germans in the second half of life. Results of a population-based observational study. BMC geriatrics. 2017;17(1):207. Epub 2017/09/10. doi: 10.1186/s12877-017-0592-3. PubMed PMID: 28886697; PubMed Central PMCID: PMCPMC5591529.

61. Han BH, Moore AA, Ferris R, Palamar JJ. Binge Drinking Among Older Adults in the United States, 2015 to 2017. Journal of the American Geriatrics Society. 2019;67(10):2139-44. Epub 2019/08/01. doi: 10.1111/jgs.16071. PubMed PMID: 31364159; PubMed Central PMCID: PMCPMC6800799.

62. Heegaard K, Avlund K, Holm-Pedersen P, Hvidtfeldt UA, Bardow A, Granbak M. Amount and type of alcohol consumption and missing teeth among community-dwelling older adults: Findings from the Copenhagen Oral Health Senior study. Journal of public health dentistry. 2011;71(4):318-26. doi: http://dx.doi.org/10.1111/j.1752-7325.2011.00276.x. PubMed PMID: 51586275.

63. Hoeck S, Van Hal G. Unhealthy drinking in the Belgian elderly population: prevalence and associated characteristics. European journal of public health. 2013;23(6):1069-75. doi: http://dx.doi.org/10.1093/eurpub/cks152. PubMed PMID: 2013-42700-012.

64. Hongthong D, Somrongthong R, Wongchaiya P, Kumar R. Factors Predictive Of Alcohol Consumption Among Elderly People In A Rural Community: A Case Study In Phayao Province Thailand. Journal of Ayub Medical College, Abbottabad : JAMC. 2016;28(2):237-40. Epub 2016/04/01. PubMed PMID: 28718548.

65. Ilomaki J, Gnjidic D, Hilmer SN, Le Couteur DG, Naganathan V, Cumming RG, et al. Psychotropic drug use and alcohol drinking in community-dwelling older Australian men: the CHAMP study. Drug and alcohol review. 2013;32(2):218-22. Epub 2012/08/14. doi: 10.1111/j.1465-3362.2012.00496.x. PubMed PMID: 22882728.

66. Ilomaki J, Gnjidic D, Le Couteur DG, Bell JS, Blyth FM, Handelsman DJ, et al. Alcohol consumption and tobacco smoking among community-dwelling older Australian men: the Concord Health and Ageing in Men Project. Australasian journal on ageing. 2014;33(3):185-92. Epub 2014/02/14. doi: 10.1111/ajag.12048. PubMed PMID: 24521471.

67. Immonen S, Valvanne J, Pitkala KH. Prevalence of at-risk drinking among older adults and associated sociodemographic and health-related factors. The journal of nutrition, health & aging. 2011;15(9):789-94. Epub 2011/11/18. PubMed PMID: 22089229.

68. Immonen S, Valvanne J, Pitkälä KH. The prevalence of potential alcohol-drug interactions in older adults. Scand J Prim Health Care. 2013;31(2):73-8. Epub 2013/04/30. doi: 10.3109/02813432.2013.788272. PubMed PMID: 23621352; PubMed Central PMCID: PMCPMC3656398.

69. Ivan MC, Amspoker AB, Nadorff MR, Kunik ME, Cully JA, Wilson N, et al. Alcohol use, anxiety, and insomnia in older adults with generalized anxiety disorder. The American journal of geriatric psychiatry : official journal of the American Association for Geriatric Psychiatry. 2014;22(9):875-83. Epub 2013/08/27. doi: 10.1016/j.jagp.2013.04.001. PubMed PMID: 23973253; PubMed Central PMCID: PMCPMC3842378.

70. Jentsch F, Allen J, Fuchs J, von der Lippe E. Typical patterns of modifiable health risk factors (MHRFs) in elderly women in Germany: results from the cross-sectional German Health Update (GEDA) study, 2009 and 2010. BMC women's health. 2017;17:1-10. doi: 10.1186/s12905-017-0380-4. PubMed PMID: 122334120.

71. Johannessen A, Engedal K, Larsen M, Lillehovde E, Stellander LT, Helvik A-S. Alcohol and prescribed psychotropic drug use among patients admitted to a department of old-age psychiatry in Norway. Nordic Studies on Alcohol and Drugs. 2017;34(1):57-71.

72. Kim SA, Kim E, Morris RG, Park WS. Exploring the non-linear relationship between alcohol consumption and depression in an elderly population in Gangneung: the Gangneung Health Study. Yonsei medical journal. 2015;56(2):418-25. Epub 2015/02/17. doi: 10.3349/ymj.2015.56.2.418. PubMed PMID: 25683990; PubMed Central PMCID: PMCPMC4329353.

73. Kim JW, Byun MS, Yi D, Lee JH, Ko K, Jeon SY, et al. Association of moderate alcohol intake with in vivo amyloid-beta deposition in human brain: A cross-sectional study. PLoS medicine. 2020;17(2):e1003022. Epub 2020/02/26. doi: 10.1371/journal.pmed.1003022. PubMed PMID: 32097439; PubMed Central PMCID: PMCPMC7041799.

74. Kohno K, Niihara H, Hamano T, Takeda M, Nakagawa Y, Shiwaku K, et al. J-curve association between alcohol intake and varicose veins in Japan: The Shimane CoHRE Study. The Journal of dermatology. 2019;46(10):902-6. Epub 2019/07/30. doi: 10.1111/1346-8138.15022. PubMed PMID: 31353631.

75. Lasebikan VO, Gureje O. Lifetime and 7-day alcohol consumption in the elderly, prevalence and correlates: Reports from the Ibadan Study of Aging. African journal of medicine and medical sciences. 2015;44(1):33-41. Epub 2015/11/10. PubMed PMID: 26548114.

76. Li J, Wu B, Selbæk G, Krokstad S, Helvik AS. Factors associated with consumption of alcohol in older adults - a comparison between two cultures, China and Norway: the CLHLS and the HUNT-study. BMC geriatrics. 2017;17(1):172. Epub 2017/08/02. doi: 10.1186/s12877-017-0562-9. PubMed PMID: 28760157; PubMed Central PMCID: PMCPMC5537928.

77. Li J, Wu B, Tevik K, Krokstad S, Helvik AS. Factors associated with elevated consumption of alcohol in older adults-comparison between China and Norway: the CLHLS and the HUNT Study. BMJ open. 2019;9(8):e028646. Epub 2019/08/05. doi: 10.1136/bmjopen-2018-028646. PubMed PMID: 31377703; PubMed Central PMCID: PMCPMC6687031.

78. Lima MC, Simao MO, Oliveira JB, Cavariani MB, Tucci AM, Kerr-Correa F. Alcohol use and falls among the elderly in Metropolitan Sao Paulo, Brazil. Cadernos de saude publica. 2009;25(12):2603-11. Epub 2010/03/02. PubMed PMID: 20191151.

79. Listabarth S, Vyssoki B, Waldhoer T, Gmeiner A, Vyssoki S, Wippel A, et al. Hazardous alcohol consumption among older adults: A comprehensive and multi-national analysis of predictive factors in 13,351 individuals. Eur Psychiatry. 2020;64(1):e4. Epub 2020/12/22. doi: 10.1192/j.eurpsy.2020.112. PubMed PMID: 33342458; PubMed Central PMCID: PMCPMC8057428.

80. Machado MPA, Opaleye DC, Pereira TV, Padilla I, Noto AR, Prince M, et al. Alcohol and tobacco consumption concordance and its correlates in older couples in Latin America. Geriatrics & gerontology international. 2017;17(11):1849-57. Epub 2017/01/07. doi: 10.1111/ggi.12974. PubMed PMID: 28060438; PubMed Central PMCID: PMCPMC5724508.

81. Marti CN, Choi NG, DiNitto DM, Choi BY. Associations of lifetime abstention and past and current alcohol use with late-life mental health: a propensity score analysis. Drug and alcohol dependence. 2015;149:245-51. Epub 2015/03/03. doi: 10.1016/j.drugalcdep.2015.02.008. PubMed PMID: 25725932.

82. McClure LA, Fernandez CA, Clarke TC, Leblanc WG, Arheart KL, Fleming LE, et al. Risky drinking in the older population: a comparison of Florida to the rest of the US. Addictive behaviors. 2013;38(4):1894-7. Epub 2013/02/06. doi: 10.1016/j.addbeh.2012.12.020. PubMed PMID: 23380494.

83. Moore AA, Karno MP, Grella CE, Lin JC, Warda U, Liao DH, et al. Alcohol, tobacco, and nonmedical drug use in older U.S. Adults: data from the 2001/02 national epidemiologic survey of alcohol and related conditions. Journal of the American Geriatrics Society. 2009;57(12):2275-81. Epub 2009/10/31. doi: 10.1111/j.1532-5415.2009.02554.x. PubMed PMID: 19874409; PubMed Central PMCID: PMCPMC3646625.

84. Muñoz M, Ausín B, Santos-Olmo AB, Härter M, Volkert J, Schulz H, et al. Alcohol use, abuse and dependence in an older European population: Results from the MentDis_ICF65+ study. PloS one. 2018;13(4):e0196574. Epub 2018/05/01. doi: 10.1371/journal.pone.0196574. PubMed PMID: 29708993; PubMed Central PMCID: PMCPMC5927409.

85. Nadkarni A, Acosta D, Rodriguez G, Prince M, Ferri CP. The psychological impact of heavy drinking among the elderly on their co-residents: the 10/66 group population based survey in the Dominican Republic. Drug and alcohol dependence. 2011;114(1):82-6. Epub 2010/10/26. doi: 10.1016/j.drugalcdep.2010.09.005. PubMed PMID: 20970926; PubMed Central PMCID: PMCPMC3123469.

86. Nogueira EL, Neto AC, Cauduro MHF, Ulrich LEF, Spanemberg L, DeCarli GA, et al. Prevalence and patterns of alcohol misuse in a community-dwelling elderly sample in Brazil. Journal of aging and health. 2013;25(8):1340-57. doi: http://dx.doi.org/10.1177/0898264313506461. PubMed PMID: 2014-00121-005.

87. Nuevo R, Chatterji S, Verdes E, Naidoo N, Ayuso-Mateos JL, Miret M. Prevalence of alcohol consumption and pattern of use among the elderly in the WHO European Region. European addiction research. 2015;21(2):88-96. Epub 2014/11/22. doi: 10.1159/000360002. PubMed PMID: 25413452.

88. Parikh RB, Junquera P, Canaan Y, Oms JD. Predictors of binge drinking in elderly Americans. The American journal on addictions. 2015;24(7):621-7. Epub 2015/08/25. doi: 10.1111/ajad.12275. PubMed PMID: 26300301.

89. Rao R, Schofield P, Ashworth M. Alcohol use, socioeconomic deprivation and ethnicity in older people. BMJ open. 2015;5(8):e007525. Epub 2015/08/26. doi: 10.1136/bmjopen-2014-007525. PubMed PMID: 26303334; PubMed Central PMCID: PMCPMC4550718.

90. Roson B, Monte R, Gamallo R, Puerta R, Zapatero A, Fernandez-Sola J, et al. Prevalence and routine assessment of unhealthy alcohol use in hospitalized patients. European journal of internal medicine. 2010;21(5):458-64. Epub 2010/09/08. doi: 10.1016/j.ejim.2010.04.006. PubMed PMID: 20816605.

91. Ryan M, Merrick EL, Hodgkin D, Horgan CM, Garnick DW, Panas L, et al. Drinking patterns of older adults with chronic medical conditions. Journal of general internal medicine. 2013;28(10):1326-32. Epub 2013/04/24. doi: 10.1007/s11606-013-2409-1. PubMed PMID: 23609178; PubMed Central PMCID: PMCPMC3785666.

92. Sacco P, Bucholz KK, Spitznagel EL. Alcohol use among older adults in the National Epidemiologic Survey on Alcohol and Related Conditions: a latent class analysis. Journal of studies on alcohol and drugs. 2009;70(6):829-38. Epub 2009/11/10. doi: 10.15288/jsad.2009.70.829. PubMed PMID: 19895759; PubMed Central PMCID: PMCPMC2776115.

93. Sanford NN, Sher DJ, Xu X, Ahn C, D'Amico AV, Aizer AA, et al. Alcohol Use Among Patients With Cancer and Survivors in the United States, 2000-2017. J Natl Compr Canc Netw. 2020;18(1):69-79. Epub 2020/01/08. doi: 10.6004/jnccn.2019.7341. PubMed PMID: 31910381.

94. Satre DD, Sterling SA, Mackin RS, Weisner C, Satre DD, Sterling SA, et al. Patterns of alcohol and drug use among depressed older adults seeking outpatient psychiatric services. American Journal of Geriatric Psychiatry. 2011;19(8):695-703. doi: 10.1097/JGP.0b013e3181f17f0a. PubMed PMID: 108245461.

95. Suo C, Yang Y, Yuan Z, Zhang T, Yang X, Qing T, et al. Alcohol Intake Interacts with Functional Genetic Polymorphisms of Aldehyde Dehydrogenase (ALDH2) and Alcohol Dehydrogenase (ADH) to Increase Esophageal Squamous Cell Cancer Risk. J Thorac Oncol. 2019;14(4):712-25. Epub 2019/01/15. doi: 10.1016/j.jtho.2018.12.023. PubMed PMID: 30639619.

96. Tevik K, Selbaek G, Engedal K, Seim A, Krokstad S, Helvik AS. Use of alcohol and drugs with addiction potential among older women and men in a population-based study. The Nord-Trondelag Health Study 2006-2008 (HUNT3). PloS one. 2017;12(9):e0184428. Epub 2017/09/09. doi: 10.1371/journal.pone.0184428. PubMed PMID: 28886172; PubMed Central PMCID: PMCPMC5590962.

97. Towers A, Philipp M, Dulin P, Allen J. The "Health Benefits" of Moderate Drinking in Older Adults may be Better Explained by Socioeconomic Status. The journals of gerontology Series B, Psychological sciences and social sciences. 2018;73(4):649-54. Epub 2016/12/09. doi: 10.1093/geronb/gbw152. PubMed PMID: 27927745.

98. Towers A, Szabó Á, Newcombe DAL, Sheridan J, Moore AA, Hyde M, et al. Hazardous Drinking Prevalence and Correlates in Older New Zealanders: A Comparison of the AUDIT-C and the CARET. Journal of aging and health. 2019;31(10):1770-89. Epub 2018/08/28. doi: 10.1177/0898264318794108. PubMed PMID: 30145918; PubMed Central PMCID: PMCPMC6393211.

99. Vafeas C, Graham R, de Jong G, Sharp J, Ngune I, Maes S. Alcohol consumption patterns of older adults: a study in a regional town in Western Australia. Contemporary nurse. 2017;53(6):647-57. Epub 2017/12/28. doi: 10.1080/10376178.2017.1421051. PubMed PMID: 29279033.

100. Villar Luis MA, de Lima Garcia MV, Pinto Barbosa S, da Costa Lima DW. Use of alcohol among elderly people attending Primary Health Care. Acta Paulista de Enfermagem. 2018;31(1):46-53. doi: 10.1590/1982-0194201800008. PubMed PMID: 129621100.

101. Waern M, Marlow T, Morin J, Ostling S, Skoog I. Secular changes in at-risk drinking in Sweden: birth cohort comparisons in 75-year-old men and women 1976-2006. Age and ageing. 2014;43(2):228-34. Epub 2013/09/27. doi: 10.1093/ageing/aft136. PubMed PMID: 24067499; PubMed Central PMCID: PMCPMC3927771.

102. Wang S, Ungvari GS, Forester BP, Chiu HFK, Wu Y, Kou C, et al. Gender differences in general mental health, smoking, drinking and chronic diseases in older adults in Jilin province, China. Psychiatry research. 2017;251:58-62. Epub 2017/02/12. doi: 10.1016/j.psychres.2017.02.007. PubMed PMID: 28189080.

103. Weyerer S, Schaufele M, Eifflaender-Gorfer S, Kohler L, Maier W, Haller F, et al. At-risk alcohol drinking in primary care patients aged 75 years and older. International journal of geriatric psychiatry. 2009;24(12):1376-85. Epub 2009/04/22. doi: 10.1002/gps.2274. PubMed PMID: 19382111.

104. Wilson SR, Knowles SB, Huang Q, Fink A. The prevalence of harmful and hazardous alcohol consumption in older U.S. adults: data from the 2005-2008 National Health and Nutrition Examination Survey (NHANES). Journal of general internal medicine. 2014;29(2):312-9. Epub 2013/10/09. doi: 10.1007/s11606-013-2577-z. PubMed PMID: 24101531; PubMed Central PMCID: PMCPMC3912311.

105. Zaitsu M, Takeuchi T, Kobayashi Y, Kawachi I. Light to moderate amount of lifetime alcohol consumption and risk of cancer in Japan. Cancer. 2020;126(5):1031-40. Epub 2019/12/10. doi: 10.1002/cncr.32590. PubMed PMID: 31814116; PubMed Central PMCID: PMCPMC7027900.
