# Supplementary material for: A systematic review of self-report measures used in epidemiological studies to assess alcohol consumption among older adults
Source: PLoS One. 2021 Dec 16;16(12):e0261292. doi: 10.1371/journal.pone.0261292 (PMC8675766; doi:10.1371/journal.pone.0261292)
Supplement: Attachment — Submitted filename: PLOS One review.docx [file pone.0261292.s003.docx]

PLOS One rev.

This is an important systematic review of alcohol consumption and pattern measurement for epidemiological studies of older adults (65 plus years old). The review including 105 studies winnowed from many more in a comprehensive search found wide variation in every aspect of measurement strategy and importantly alcohol use pattern definitions. Basic variance was also found in drink size included, duration of measurement and type of measure, i.e., QF, BSQF, GQF Weekly diary, Last occasion. Conclusions and implications proposed by the authors emphasized the need for standardization. The manuscript is very well written and provides a wealth of tabular material.

I have a number of comments and suggestions for improvement of the paper:

1) LL 104-113 The list of measurement approaches is fine but there is here and in many places following an overuse of secondary references 24 and 36, (WHO international measurement guidelines and SMART, respectively). While I have nothing against these citations based essentially of expert groups, some other citations could help reduce the over-reliance one example being Greenfield & Kerr, Alcohol measurement methodology in epidemiology: recent advances and opportunities- Addiction, 2008. It would be worth emphasizing that last drinking occasion (and yesterday drinking especially) typically have specialized uses for better calibrating longer duration measures such as 12-month measures (e.g., Stockwell et al. What did you drink yesterday? Etc. Addiction, 2008).
2) I commend the authors for identifying LL 59-64 identifying perennial problems of measurement and pattern definition. A type of pattern definition that does not appear to have been given sufficient attention in the excellent review (perhaps because the older age literature has attended to it less than in general population studies), is that of relative binging vs spacing of drinks to obtain the same volume. At the lower weekly levels such as those recommended for older adults by NIAAA 5/4+ (M/W) drinks or (5 or 4 x 12 g = 60 or 40 g ethanol) is a good indicator of this intake variability. At the higher volume levels 8+ may separate spacers (or even drinkers) from bunched (or binge) drinking patterns (per the GQF measure used, however, in only one of the included studies: Satre et al. [53]). Some recommendation for a smaller set of items have noted the value of including maximum in any day (usually categorized).
3) LL 133-137 The lack of standardization – aim of a systematic review somewhat puts the cart before the hose, in the sense that the ultimate finding that there is very little standardization seems to motivate the review that finds this. The conclusions and Implications sections echo the hope for moving toward more standardization, but I think it might be acknowledges somewhere that different aims (as well as traditions and simple research group preferences) result in the variety of measures and definitions seen. The authors could note (relevant although not specific to assessing drinking patterns among older adults) that over the years there have been expert meetings and groups convened to discuss alcohol measurement and patterns, and sometimes to make some consensus recommendations. One meeting organized by Stockwell was in Freemantle AU, and led to reference 24. Another was at NIAAA (See Alcohol Clin Exp Res 22, 4S-14S & 52S-62S, 1998). A third was a conference organized by Room held at Skarpö, Sweden in 2000, resulting in a special edition of the J Substance Use (e.g., Dawson & Room, Toward agreement on ways to measure and report drinking patterns and alcohol-related problems in adult general population surveys: the Skarpö Conference overview. J Subst Abuse 12, 1-21, 2000. SMART [Ref 36] represents a more recent effort. I therefore feel that the conclusions regarding standardization might be tempered by acknowledging the many previous such efforts of groups of alcohol epidemiologists, which have thus far failed to fully achieve such a standardization of alcohol measures and pattern definitions for general population surveys and similarly by extension for the aging subgroup. However, the authors are commended for recognizing that patterns and guidelines could differ in the elder group, and that on guidelines there is no empirically backed international guideline (LL 542-543).
4) LL 483-484 An important reference that complements [134] is Rehm, et al. Assessment methods for alcohol consumption, prevalence of high risk drinking and harm: a sensitivity analysis. Int J Epidemiol 28, 219-224, 1999. Even though GQF was only used in one paper in the review, this proved superior to QF and weekly diary in capturing risky and harmful drinking volumes.
