# Supplementary material for: A systematic review of self-report measures used in epidemiological studies to assess alcohol consumption among older adults
Source: PLoS One. 2021 Dec 16;16(12):e0261292. doi: 10.1371/journal.pone.0261292 (PMC8675766; doi:10.1371/journal.pone.0261292)
Supplement: Attachment — Submitted filename: Comments.docx [file pone.0261292.s004.docx]

Dr. Tevik and collaborators reported in an elegant systematic review how different are the parameters used for the categorization/assessment of alcohol drinking among epidemiological studies. This paper raised an important question since the assessment of alcohol consumption is used for the diagnostic of alcohol use disorder, thus if studies/hospitals don’t use the same criteria and definitions the diagnostics could be under or overestimated. All the criteria used for the studies selection/inclusion were well thinking and organized. Authors also followed a recommended structure for systematic review (prism9). Data is well reported and clear in all tables throughout the manuscript. All important “side” points were mentioned, such as problems with stigma; underestimation of alcohol consumption during self-report; the population’s knowledge about the definitions of standard-drinking and the use of figures to clarify that during interviews. The limitations and strengths were also mentioned. My two suggestions are: 1) Flip the title: A systematic review of self-report measures used in epidemiological studies to assess alcohol consumption among older adults; 2) Although authors included the country of each study on table 3, would be also interesting if they keep that information on table 6, so the readers would have an idea about which are the criteria used in each country. Besides that, the study is timely, the methodology applied is sound, the authors provide clear models and hypothesis, and the findings are clearly embedded in a theoretical framework in the discussion section.
